# Redox‐Programmed Activation of a Dual‐Modal Probe for High‐Fidelity Tumor Delineation and Image‐Guided Surgery

**DOI:** 10.1002/advs.202519223

**Published:** 2026-01-04

**Authors:** Haohao Yan, Yanbin Feng, Qian Jia, Ruili Zhang, Zhongliang Wang

**Affiliations:** ^1^ Lab of Molecular Imaging and Translational Medicine (MITM) Engineering Research Center of Molecular & Neuroimaging Ministry of Education School of Life Science and Technology Xidian University Xi'an Shaanxi China

**Keywords:** activatable probe, imaging‐guided tumor surgery, molecular imaging, tumor detection

## Abstract

The nonspecific activation of activatable probes presents significant challenges in their applications for accurate cancer detection, leading to false signals in normal tissues and the potential oversight of microlesions. To address this issue, we developed a glutathione (GSH)‐activatable magnetic resonance imaging (MRI) and near‐infrared II (NIR‐II) fluorescent probe (GAP9) using a redox capacity engineering strategy. By systematically adjusting the reaction pH during probe synthesis, we could precisely modulate its oxidation capacity to ensure that the activation window of the probe precisely matched tumor GSH concentrations. This strategy ensures that GAP9 remains in the “OFF” state within normal tissues through dual MRI/NIR‐II quenching mechanisms, minimizing false‐positive signals and background noise. Upon reaching tumor sites, GAP9 undergoes GSH‐triggered disassembly, rapidly activating T1‐weighted MRI for preoperative tumor mapping and unlocking NIR‐II fluorescence for real‐time intraoperative tumor delineation. This tumor‐adaptable strategy enables the specific localization of microtumor lesions, intraoperative margin monitoring, and complete excision of ultrasmall residual foci ≤1 mm, achieving a 96% detection rate in a mouse model of peritoneal metastasis. This study presents a novel paradigm in molecular probe design, emphasizing the potential of integrating programmable redox chemistry with tumor‐specific characteristics to enhance detection accuracy, ultimately improving surgical outcomes and patient prognoses.

## Graphical abstract

This study presents a glutathione (GSH)‐activatable dual‐modality probe (GAP9) with a redox‐tuned activation window for precise tumor detection. It remains quenched (“OFF”) in normal tissue yet activates specifically in tumors for T1‐weighted magnetic resonance imaging (MRI) preoperative mapping and near‐infrared II (NIR‐II) fluorescence intraoperative guidance, achieving >95% detection rate and complete resection of sub‐millimeter lesions.

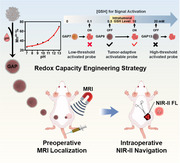

## 1 Introduction

Accurate intraoperative delineation and removal of microtumor lesions present significant challenges in cancer treatment [1], increasing the risk of tumor recurrence and metastasis owing to limitations in diagnostic sensitivity and surgical precision [2]. Despite progress in spatiotemporal resolution [3], conventional single‐modality imaging continues to face inherent trade‐offs concerning imaging penetration depth [4], functional specificity [5], and the capability to provide real‐time surgical guidance [6]. These restrictions are particularly problematic when addressing early‐stage and micrometastatic tumors [7].

Integrating multimodal imaging approaches has emerged as a promising strategy to address these challenges [8]. For example, magnetic resonance imaging (MRI) is a valuable tool for preoperative tumor localization [9], providing high‐resolution anatomical information that is essential for surgical planning [10]. Fluorescence imaging [11], especially within the second near‐infrared window (NIR‐II, 1000–1700 nm) [12], excels in intraoperative settings because of its high sensitivity [13], real‐time visualization [14], and ability to provide immediate feedback [15], which are critical for precise tumor resection [16]. The synergistic integration of MRI for preoperative localization and fluorescence imaging for intraoperative guidance seeks to exploit the advantages of both modalities, thereby facilitating a comprehensive evaluation for accurate tumor treatment [17]. Nevertheless, the clinical application of multimodal probes is frequently impeded by persistent “always‐on” signaling mechanisms [18]. Such signaling may result in false‐positive detections in healthy tissues [19], posing a substantial challenge in accurately targeting the molecular characteristics associated with tumors [20].

Glutathione (GSH) plays a vital role in various cellular processes, such as detoxification, antioxidant defense, and the maintenance of redox homeostasis [21]. Tumor cells often exhibit higher levels of GSH (0.5–10 mm) than normal cells, which makes GSH a promising target for developing tumor‐specific imaging probes and therapeutic strategies [22]. However, current GSH‐activatable probes suffer from rigid activation thresholds due to their inflexible chemical structures [23]. Probes based on thiol‐disulfide equilibrium systems and metal‐ligand coordination exhibit excessive sensitivity to physiological concentrations of GSH in healthy organs [24]. On the other hand, manganese oxide nanoparticles (MONs)‐based probes, synthesized through a one‐step in situ redox reaction using potassium permanganate and reducing agents, require higher thresholds for GSH activation (≥10 mm) [25], increasing the risk of missing microtumor foci. This binary “all‐or‐nothing” responsiveness may lead to clinically unacceptable errors such as false‐positive signals and the failure to detect microtumors [26]. Consequently, the development of novel activatable imaging probes that can accurately match the tumor‐specific GSH concentration range is highly desirable.

**SCHEME 1:**
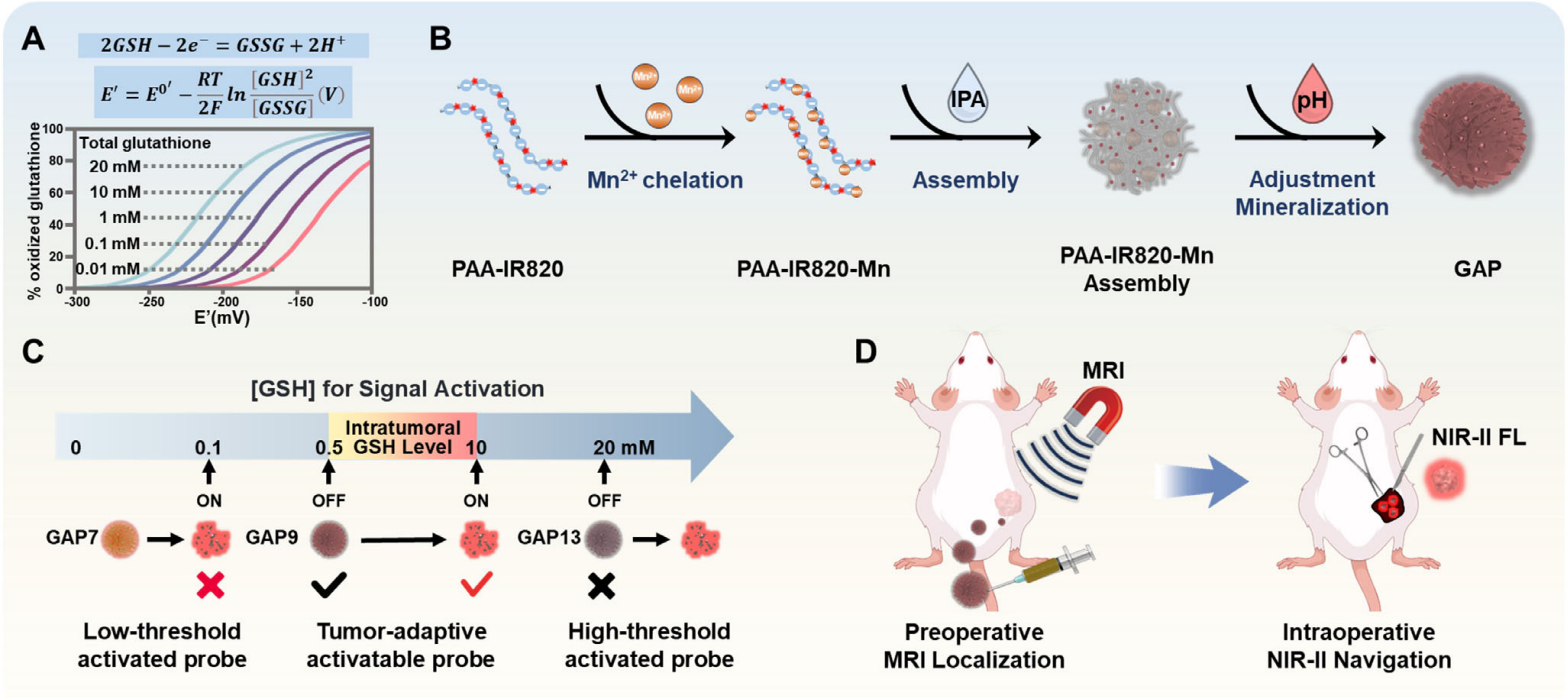
The schematic diagram of GAP. (A) Correlation between the half‐cell reduction potential *E′* and the percentage of oxidized glutathione. The equilibrium between GSSG and GSH can be calculated using the Nernst equation, resulting in sigmoidal *E′*‐GSSG diagrams. *E′* not only depends on the [GSH]/[GSSG] ratio but also on the indicated total concentration of glutathione (as emphasized in the upper version of the Nernst equation). An increase of glutathione—e.g., due to the de novo biosynthesis or uptake of GSH—shifts the curve to the left. (B) Schematic illustration of a GSH‐responsive GAP system for tumor‐specific activation. (C) Schematic of tumor‐specific activation mechanism for GAP. (D) Schematic illustration of GAP for preoperative MRI tumor localization and intraoperative NIR‐II fluorescence aavigation.

This “threshold dilemma” arises from treating GSH solely as a chemical reductant, thereby neglecting its role as a concentration‐dependent regulator in the redox equilibrium between GSH and its oxidized form, glutathione disulfide (GSSG) [27]. This conversion establishes an electrochemical gradient in biological systems, where the redox potential (*E*) of the GSH/GSSG pair depends on the total concentration of GSH as exemplified by the Nernst equation [28]. It has been demonstrated that increased GSH shifts the *E* curve to more reducing values (Scheme 1A). Inspired by this concentration‐encoded redox signature, we hypothesized that the reduction capacity of GSH in tumors differs from that in normal tissues, as GSH levels are elevated in tumor tissues. By designing a probe whose reactivity is precisely calibrated to this tumor‐elevated reducing capacity, we may establish a tumor‐specific activation window to discriminate between tumors and normal tissue.

Herein, we propose a redox capacity engineering strategy to fabricate a series of GSH‐activatable MRI/NIR‐II fluorescent probes (GAPs) with varied GSH‐responsive ranges, through polyacrylic acid (PAA)‐mediated co‐assembly of MONs and IR820 iodide. By systematically adjusting the reaction pH (7.0–13.0), the redox capacity of the GAP can be precisely controlled. This modulation allows for the decoupling of the probe's chemical structure from its activation behavior, establishing a tumor‐specific GSH‐activation window. Under physiological conditions and in normal tissues, the GAP functions through a dual‐quenching mechanism. The stable confinement of Mn ions within the nanoparticles diminishes their transverse relaxation capability, thereby suppressing the T1‐weighted MRI signal. Simultaneously, IR820 experiences aggregation‐caused quenching (ACQ) of NIR‐II fluorescence, maintaining an “OFF” state. However, upon reaching tumor sites, GAP rapidly disassembles in response to GSH, which initiates the reduction of Mn^4+^ to Mn^2+^. The Mn^2+^ subsequently binds to serum proteins, enhancing longitudinal relaxation and switching MRI to an “ON” state for preoperative microlesion mapping. Meanwhile, the liberated IR820 restores NIR‐II emission (∼1050 nm) for real‐time intraoperative delineation of tumor margins. Thanks to this redox capacity engineering, the GAP probe can be specifically activated within the tumor GSH range while remaining inactive in normal tissues. This resulted in a 96% tumor detection accuracy and reduced false signals. This study successfully integrates programmable redox chemistry with dual‐modal signal coordination, addressing the critical challenge of nonspecific activation that often affects conventional responsive probes and enhancing the fidelity of molecular sensing with surgical precision through tumor‐specific GSH activation.

## 2 Results and Discussion

### 2.1 Synthesis and Characterization of GAPs

MONs are biocompatible and GSH‐responsive materials, with their oxidizing capacity dependent on the valence state of Mn. Inspired by the equilibrium between GSH and GSSG (Scheme 1A), we hypothesized that the different oxidation states of MONs play a crucial role in generating a highly sensitive and specific response within a defined concentration range of GSH.

Unlike traditional MONs synthesis, we oxidized Mn^2+^ under mild air conditions at different pH levels to produce GAP probes with distinct oxidation capacities. The preparation of the GAP probes is illustrated in Scheme 1B. We first synthesized the NIR‐II cyanine dye IR820‐NH_2_ (Scheme S1 and Figure S1–S3), which was then conjugated with PAA to form PAA‐IR820. Next, MnCl_2_ was introduced into the aqueous solution of PAA‐IR820, followed by the addition of isopropanol (IPA) to facilitate the assembly of the PAA‐IR820‐Mn. The pH of the reaction system was subsequently adjusted using varying concentrations of NaOH (pH 7.0 – 13.0) to promote the mineralization of Mn^2+^ in air, yielding GAP probes with varying oxidation capacities.

The synthesis of PAA‐IR820 was initially monitored using absorption and fluorescence emission spectroscopy. As shown in Figure S4, the absorption and emission spectra of IR820‐NH_2_ and PAA‐IR820 exhibited the characteristic peaks of IR820, with NIR‐II tailing emission extending beyond 1000 nm. The formation of GAP was subsequently validated through a combination of photographs, absorption spectroscopy, hydrodynamic size analysis, and zeta potential measurements (Figure S5). As the synthesis pH increased, the color of the resulting GAPs gradually changed from light yellow to black, indicating a shift in the valence state of Mn of GAPs (Figure S5A). Furthermore, the absorption peaks observed in the 700–900 nm validated the successful encapsulation of IR820 in its aggregated quenching state (Figure S5B). Dynamic light scattering (DLS) and zeta potential measurements revealed that the synthesized GAPs had a similar size and surface charge (Figure S5C,D).

**FIGURE 1:**
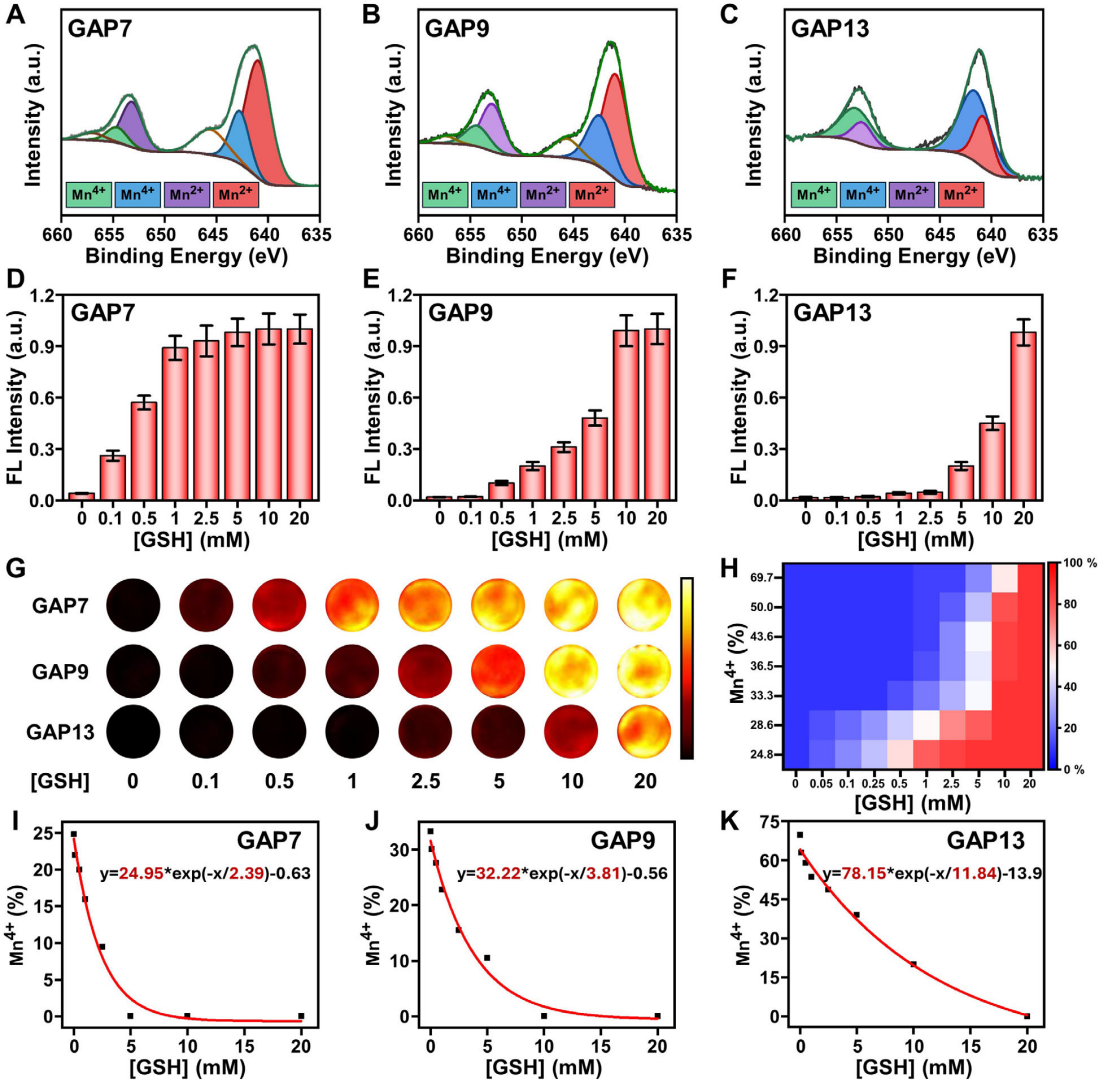
Redox capacity engineered GAPs exhibit tunable GSH response windows. (A–C) representative Mn 2p XPS spectra of GAP synthesized at pH 7, 9, and 13. (D)‐(F) GSH concentration‐dependent activation of GAP probes synthesized under different pH conditions, with [GSH] of 0.1–20 mM. All assays were conducted in phosphate‐buffered saline (PBS) at pH  7.4. The statistical analysis of fluorescence peaks at 930 nm was performed for GAP in solutions with varying GSH concentrations. All statistical data are presented as mean values ± SD. (n = 3 independent experiments). (G) GSH concentration‐dependent responses of NIR‐II imaging signals under LP1000 filter for GAP probes synthesized under different pH conditions. (H) Heatmap illustrating the recovery level of fluorescence signal for GAP probes with varying Mn^4+^ ratios across different GSH concentration ranges. (I)‐(K) GSH concentration‐dependent responses of Mn^4+^ content in GAP probes synthesized under varying pH conditions.

According to reaction Equations (1) and (2), varying concentrations of NaOH yield distinct reaction rates. This variation in reaction rates subsequently results in different proportions of Mn^2+^ to Mn^4+^ within the synthesized GAPs. Therefore, we selected GAPs synthesized at three representative pH values (pH = 7, 9, and 13), hereafter designated as GAP7, GAP9, and GAP13, respectively. X‐ray photoelectron spectroscopy (XPS) was then employed to determine the oxidation state of Mn in these particles. As shown in Figure 1A–C, the Mn^4+^ content in GAP7, GAP9, and GAP13 was determined to be 24.8%, 33.3%, and 69.7%, respectively. The Mn 2p XPS spectra for the remaining GAP samples are presented in Figure S6. These results demonstrated a positive correlation between increased synthetic pH levels and Mn^4+^ content in GAP, suggesting that the oxidizing capacity of GAP is enhanced with higher pH levels during the synthesis process (Figure S7). In addition, the Mn 3s XPS spectra confirmed that GAP synthesized under more alkaline conditions exhibited a reduced splitting energy gap (Δ*E*), indicative of a higher valence state of Mn ions (Figure S8).

(1) $$Mn^{2+}+2OH^{-}\to \mathrm{Mn}{\left(\mathrm{OH}\right)}_{2}$$

(2) $$2\mathrm{Mn}{\left(\mathrm{OH}\right)}_{2}+O_{2}\to 2\mathrm{MnO}{\left(\mathrm{OH}\right)}_{2}\to 2\mathrm{Mn}O_{2}\cdot H_{2}O$$

In our probe design, GAP encapsulates IR820 dyes, which are initially non‐emissive due to the ACQ effect. Upon interaction with GSH, GAP disassembles, releasing the encapsulated dyes and restoring fluorescence. Consequently, the extent of fluorescence recovery was directly correlated with the response of GAP to GSH. To further investigate the GSH‐responsive range of GAP synthesized under varying pH conditions, GAP probes at identical concentrations were dispersed in PBS with different GSH concentrations, and their fluorescence spectra were evaluated (Figure 1D–F). The resulting signals were normalized against the fluorescence intensity of GAP at the completion of the response. As shown in Figure 1D, GAP7 initiated its response at a low GSH concentration of 0.1 mm and achieved full activation at approximately 1 mm GSH. In contrast, GAP13 commenced its response at a significantly higher GSH concentration of 5 mm, reaching completion at 20 mm (Figure 1F). The response ranges of both GAP7 and GAP13 do not correspond with the GSH concentrations commonly found in tumor tissues. Notably, GAP9 initiated its response at 0.5 mm GSH and achieved full activation at 10 mm GSH. This range precisely matches the GSH concentrations observed in tumors (Figure 1E). The NIR‐II fluorescence images of the GAPs (Figure 1G) and the heatmap of fluorescence intensity (Figure 1H) further supported that GAP9, which contained an optimal amount of Mn^4+^, exhibited a marked change in emission at GSH concentrations found at the tumor sites, while remaining inactive at physiological levels.

To elucidate the mechanisms underlying the distinct GSH‐responsive behaviors, we analyzed XPS evolution of GAP7, GAP9, and GAP13 upon exposure to increasing concentrations of GSH (Figures S9–S11). The Mn^4+^ fraction, as determined through curve‐fitting, was plotted against the GSH concentration to precisely reflect the Mn^4+^ valence‐state transition at varying GSH concentrations. As shown in Figure 1I–K, the responsiveness to GSH was closely correlated with the initial Mn^4+^ content in GAP. The gradual addition of GSH promoted the reduction of Mn^4+^ to Mn^2+^. However, the concentration required for complete conversion differed among the three GAPs. Consistent with the fluorescence result, the GSH‐responsive ranges of GAP7 and GAP13 did not precisely match the GSH concentration relevant to the tumor (0.5–10 mm). In contrast, GAP9 achieved this transition successfully within the specified range. This selective response highlights the potential of GAP9 to differentiate between tumor and normal tissues. Collectively, these findings highlight the significant promise of GAP9 as an imaging probe activated by GSH within tumor tissues, offering a promising strategy for enhancing the precision of tumor resection. Thus, GAP9 synthesized at pH 9.0 was selected for subsequent experiments.

### 2.2 In Vitro Assessment of the GSH Response in GAP9

In addition to a suitable pH‐response range, the fluorescence ON/OFF contrast, defined as the ratio of fluorescence intensity at GSH concentrations of 10 to 0 mm, is also crucial for achieving ultrasensitive tumor imaging. To maximize fluorescence contrast, the optimal concentration of IR820 was determined by modulating the conjugation ratio of IR820‐NH_2_ to PAA, using amino‐to‐carboxyl molar ratios of 1:250, 2:250, and 4:250.

As shown in Figure S12, when the ratio was 1:250, the presence of GSH (ON state) induced a marked fluorescence, while the absence of GSH (OFF state) led to relatively weak fluorescence quenching (Figure S12A). At a ratio of 2:250, the ON state continued to exhibit a strong fluorescence, whereas the OFF state showed more pronounced quenching (Figure S12B). At a ratio of 4:250, although the OFF state exhibited significant quenching, the ON state showed a reduced fluorescence recovery (Figure S12C). This observed variation can be attributed to the distribution of dye molecules within GAP9. At a lower ratio, the weak aggregation of the dye in the OFF state mitigates the ACQ effect, thereby reducing quenching. In contrast, enhanced dispersion in the ON state leads to increased fluorescence. At a higher ratio, greater dye aggregation in the OFF state amplifies the ACQ effect, resulting in more pronounced quenching. Additionally, due to the high dye concentration, partial aggregation occurred in the ON state, hindering fluorescence recovery. Consequently, the fluorescence contrast achieved its maximum at a ratio of 2:250, with the GAP displaying a peak contrast of 70 (Figure S12D). Thus, the 2:250 ratio was determined to be the optimal IR820 conjugation ratio for the preparation of GAP9.

**FIGURE 2:**
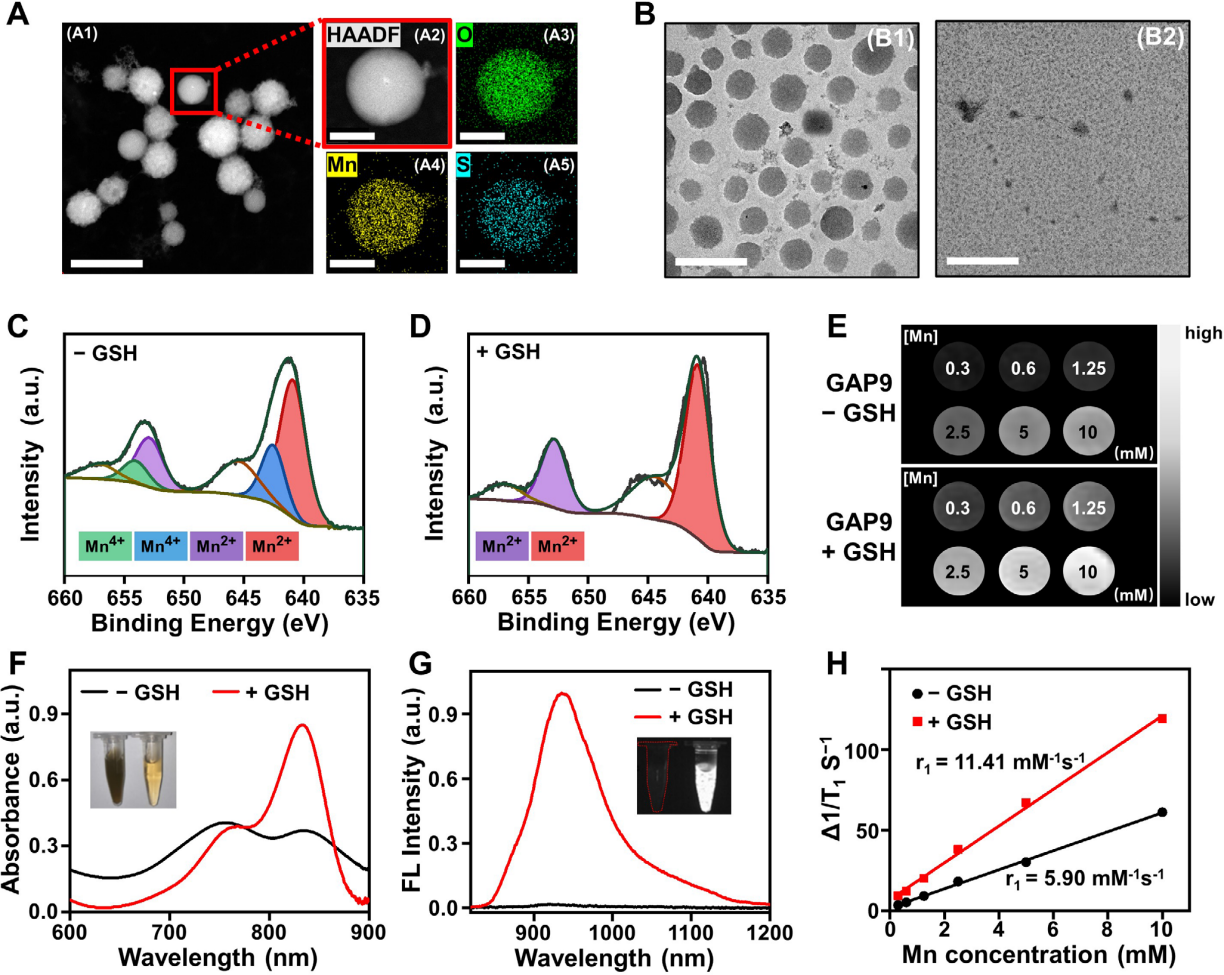
Characterization and evaluation of GSH response of GAP9. (A) Elemental mapping image of GAP9. (A1) Scale bar: 200 nm, (A2), (A3), (A4), and (A5) Scale bar: 50 nm. (B) Representative TEM images of GAP9 in PBS (B1) and PBS with GSH (10 mm) (B2). Scale bar: 200 nm. Mn 2p XPS spectra of GAP9 in (C) PBS and (D) PBS with GSH (10 mm). (E) T1‐weighted MRI of GAP9 before and after GSH activation. (F) Absorption spectra and (G) Fluorescence spectra of GAP9 in PBS (black lines) and PBS with GSH (10 mm, red lines), respectively. (H) Plot of Δ1/T1 versus Mn concentration for GAP9 in PBS (black lines) and PBS with GSH (10 mm, red lines).

To improve the biocompatibility of GAP9 for in vivo applications, we modified it with methoxy‐polyethylene glycol‐amine (m‐PEG‐NH_2_) for subsequent experiments. Successful PEGylation was confirmed by changes in the zeta potential (Figure S13A) and in the hydrodynamic size (Figure S13B). Transmission electron microscopy (TEM) image and further elemental mapping analysis in Figure 2A clearly revealed that GAP9 was uniformly composed of O (green), Mn (yellow), and S from IR820 (blue), confirming the successful formation of GAP9. The GSH‐responsive disassembly of the GAP9 was confirmed by TEM results in Figure 2B and Figure S14, which clearly showed that the spherical GAP9, with an average diameter of 80 ± 9 nm, dissociated into irregular small fragments in the presence of 10 mm GSH. This GSH responsiveness of GAP9 was supported by XPS analysis. As shown in Figure 2C, in the absence of GSH, GAP9 contained both Mn^4+^ and Mn^2+^, with Mn^4+^ accounting for approximately 33.3%. In contrast, in the presence of GSH, GAP9 was reduced and dissociated into free Mn^2+^ (Figure 2D).

Moreover, GAP9 demonstrated significantly enhanced MRI contrast capability in a solution containing GSH (10 mm) and human serum albumin (HSA, 10 mg mL^−1^), achieving a T1 relaxivity (r1) of 11.41 mm ^−1^ s^−1^ (Figure 2E). This value is substantially higher than the r1 of 5.9 mm ^−1^ s^−1^ in PBS containing HSA (Figure 2H). These results indicate that the elevated GSH levels facilitated the reduction of GAP9, leading to the release of paramagnetic Mn^2+^, which can readily interact with proteins to form a slowly rotating Mn‐protein complex, thereby significantly enhancing the MRI contrast [29]. The enhancement in MRI contrast indicates the potential application of GAP9 for GSH‐activated tumor imaging.

Consistently, the absorption and emission spectra of GAP9 showed GSH‐triggered disassembly and activation of IR820 fluorescence with a high ON/OFF contrast (Figure 2F,G). Furthermore, among the various analytes tested, only GSH at a concentration of 10 mm caused a dramatic change in the fluorescence of GAP9 under identical conditions, indicating the high specificity of GAP9 for GSH in complicated biological environments (Figures S15 and S16). The stability of GAP9 was evaluated in PBS, fetal bovine serum (FBS), medium, and medium containing 10% FBS by measuring its hydrodynamic size. As shown in Figure S17, the size of GAP9 remained nearly unchanged over 48 h, indicating its good stability in physiological media. Additionally, GAP9 exhibited negligible photobleaching (Figure S18), further demonstrating its robust stability for potential biomedical applications.

### 2.3 GAP9 Enables Highly Sensitive and Specific Imaging for In Vivo Tumor Detection

Having demonstrated the impressive GSH responsiveness of GAP9 in vitro, we proceeded to assess its GSH responsiveness at the cellular level. Before that, the cytotoxicity of GAP9 toward breast cancer cells 4T1 was evaluated. As shown in Figure S19, no measurable cytotoxicity was observed at various concentrations (up to 250 µg mL^−1^), suggesting the excellent biocompatibility of GAP9.

Given that elevated GSH levels are observed in nearly all types of solid tumors, we explored the response of GAP9 to GSH in various cancer cell lines. GAP9 was incubated with 4T1 cells, the human breast cancer cell line MCF‐7, and the human liver cancer cell line Hep G2, respectively, and NIR‐II fluorescence images were recorded. As shown in Figure S20A–C, cancer cells treated with GAP9 exhibited stronger NIR‐II fluorescence compared to those in the PBS group. However, pretreatment of the cells with N‐ethylmaleimide (NEM), a GSH scavenger, led to a notable reduction in fluorescence, indicating that GAP9 activation is dependent on the presence of GSH. Subsequent quantitative analysis consistently confirmed the GSH responsiveness of GAP9, indicating its great potential for NIR‐II tumor imaging with high specificity and sensitivity (Figure S20D).

**FIGURE 3:**
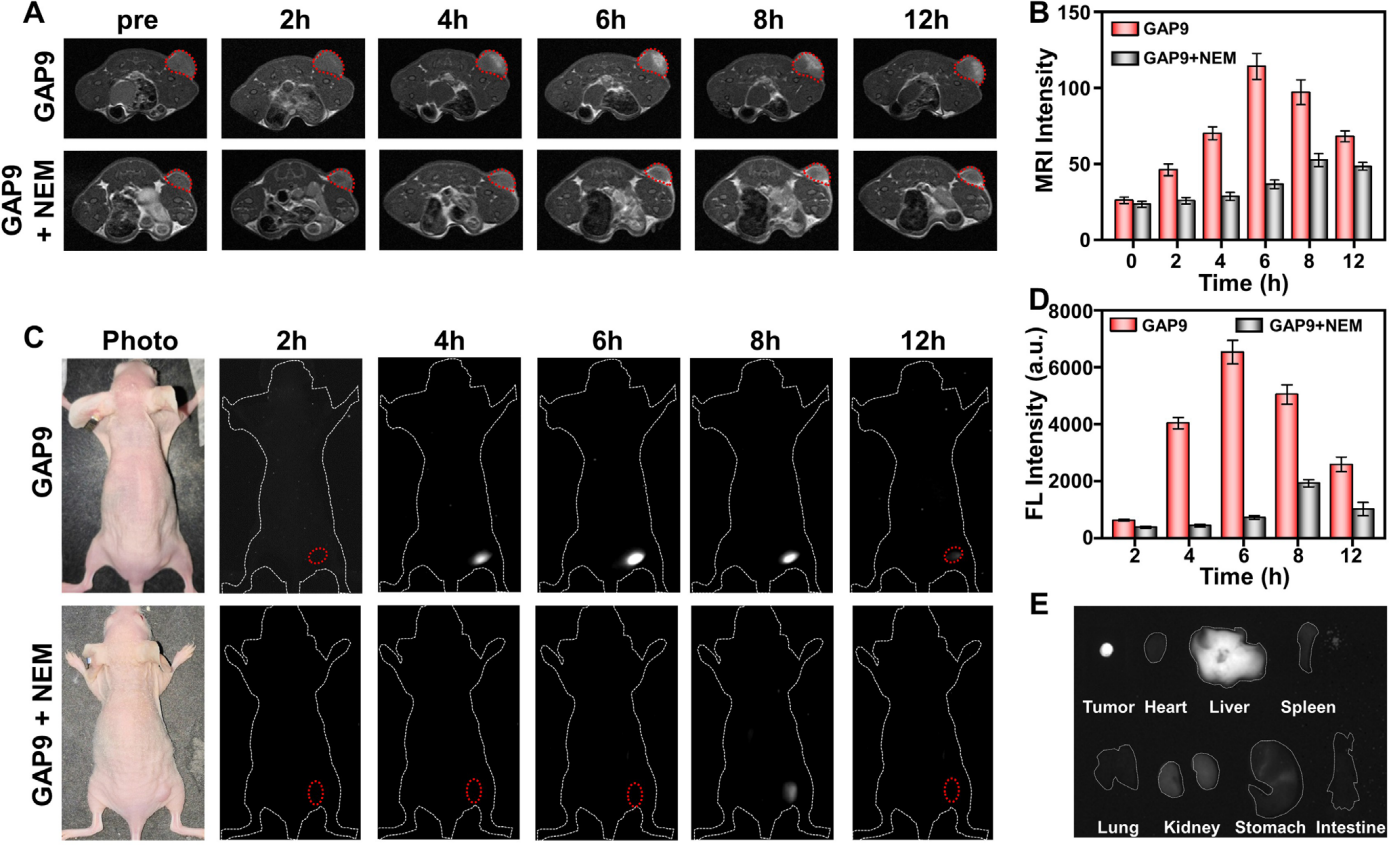
Imaging performance of GAP9 in living mice bearing subcutaneous 4T1 tumors. (A) Time‐dependent T1‐weighted MRI of 4T1 tumor‐bearing mice after intravenous injection of GAP9 under different conditions. (B) Quantitative analysis of MRI signal intensity values in tumor regions from A. Data represent the mean ± SD of triplicate measurements. (C) Time‐dependent NIR‐II fluorescence imaging of 4T1 tumor‐bearing mice after intravenous injection of GAP9 (using LP1000 filter) under different conditions. (D) Quantitative NIR‐II fluorescence signal intensity in tumor regions from C. Data represents the mean ± SD of triplicate measurements. (E) Ex vivo NIR‐II imaging of tumors and major organs collected from mice that were sacrificed post‐injection. The results were consistently reproduced in three independent mice, demonstrating the reliability of GAP9 for in vivo tumor imaging.

Encouraged by the above results, the performance of GAP9 for in vivo tumor imaging was investigated. Before that, the biosafety of GAP9 was evaluated through various physiological indicators in the blood biochemistry (Figure S21). No obvious systemic or local impairment was observed, suggesting that GAP9 can be safely utilized for in vivo applications. GAP9 was injected intravenously into mice bearing a subcutaneous 4T1 tumor, and MRI and NIR‐II fluorescence imaging were performed separately (n = 5 for each group). As shown in Figure 3A, T1‐weighted MRI signals at the tumor site gradually intensified and reached a maximum at 6 h, indicating the effective accumulation and activation of GAP9 in tumor tissues. Conversely, pretreatment with NEM markedly retarded the imaging process of GAP9 signals in the tumor area, consistent with in vitro results. The enhancement of T1 contrast was validated by the quantitative analysis shown in Figure 3B, demonstrating the feasibility of applying GAP9 for preoperative tumor localization. Similarly, NIR‐II fluorescence imaging results revealed a robust tumor detection capability of GAP9, with fluorescence signals at the tumor site progressively intensifying and reaching a peak at 6 h (Figure 3C,D). Meanwhile, there was a minimal signal observed in non‐tumor regions, greatly facilitating accurate tumor identification. The remarkable tumor recognition capability of GAP9 was supported by ex vivo imaging of the tumors and major organs collected from mice that were sacrificed post‐injection. As shown in Figure 3E and Figure S22, GAP9 exhibited higher fluorescence signals in the tumor than in other organs. This superior imaging performance of GAP9 could be probably be attributed to its specific activation by GSH within the tumor rather than normal tissues, which is crucial for implementing image‐guided tumor surgical planning and margin assessment.

To better evaluate the capability of GAP9 for real‐time tumor visualization, IR820‐loaded SiO_2_ nanoparticles (denoted as IR820@SiO_2_) were fabricated as an Always‐ON control probe for comparison (Figures S23 and S24). GAP9 and IR820@SiO_2_ were respectively injected intravenously into mice bearing 4T1 tumors of two different sizes: large and small tumors (n = 5 for each group). Fluorescence signals were collected separately within the first near‐infrared (NIR‐I) and NIR‐II windows, corresponding to the emission properties of IR820 in both regions. Although IR820@SiO_2_ allowed the imaging of a large tumor, it also produced significant non‐specific signals from normal tissues in both imaging windows (Figure S25A). In contrast, GAP9 clearly delineated the tumor with minimal background interference, especially in the NIR‐II window owing to reduced light scattering and tissue autofluorescence, demonstrating the appreciable ability of GAP9 to distinguish the tumor from normal tissues (Figure S25B). Further quantitative analysis in Figure S25C revealed that GAP9 achieved a substantially higher tumor‐to‐normal tissue (T/N) ratio than the Always‐ON probe IR820@SiO_2_ (12 versus 2).

The remarkable imaging capability of GAP9 was demonstrated in small tumor imaging. As shown in Figure S25D, IR820@SiO_2_ failed to identify small tumors, with no detectable fluorescence signal observed in either imaging window. In contrast, GAP9 successfully localized a small tumor as a bright spot (Figure S25E) and achieved a high T/N ratio of 12 within the NIR‐II imaging window (Figure S25F). Moreover, the T/N ratios for all mice were consistently higher in the NIR‐II window compared to the NIR‐I window, further highlighting the advantages of NIR‐II imaging. The tumor size was confirmed through ex vivo images and hematoxylin and eosin (H&E) staining results, as presented in Figure S26. Collectively, these findings underscore the superior tumor imaging capabilities of GAP9, even in small tumor models (≤1 mm), and emphasize the benefits of NIR‐II fluorescence imaging for enhanced tumor visualization and imaging‐guided surgery.

### 2.4 GAP9 Facilitates Margin‐clear Tumor Resection Via Intraoperative NIR‐II Fluorescence Imaging

**FIGURE 4:**
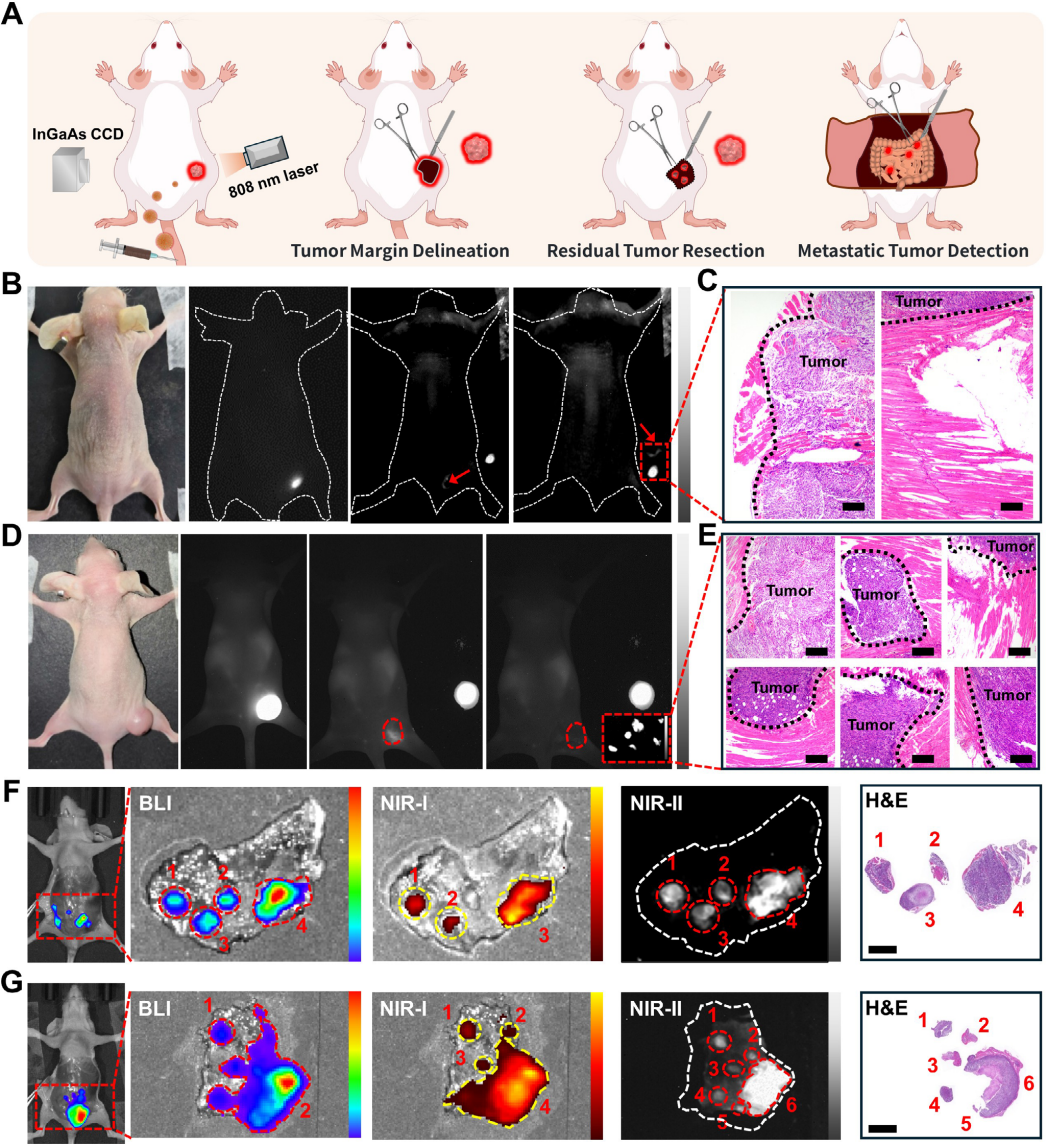
NIR‐II imaging‐guided surgery by GAP9. (A) Schematic diagram of NIR‐II imaging‐guided intraoperative delineation for surgical margins, residual tumors, and metastatic lesions. (B) NIR‐II imaging‐guided surgical margin clearance after intravenous injection of GAP9. (C) H&E staining of margin lesions identified from (B). Scale bars: 0.2 mm. (D) NIR‐II imaging‐guided surgical resection of residual tumors after intravenous injection of GAP9. (E) H&E staining of residual tumor tissues excised based on (D). Scale bars: 0.2 mm. (F) ‐ (G) Multimodal comparison (BLI, NIR‐I, NIR‐II, and H&E staining) for detecting peritoneal metastatic tumors after intravenous injection of GAP9. Scale bars: 2 mm. The results were consistently reproduced in at least three independent mice, demonstrating the reliability of GAP9 for in vivo NIR‐II fluorescence‐guided surgery.

The exceptional tumor recognition capability of GAP9 motivated us to explore its potential application in NIR‐II fluorescence‐guided surgery. To this end, we designed experiments to simulate tumor margin delineation and the resection of residual foci, which are critical for successful tumor removal and improved patient prognosis (Figure 4A). GAP9 was intravenously injected into mice bearing a 4T1 tumor 6 h prior to surgery, and tumor resection was conducted under the guidance of NIR‐II imaging. As shown in Figure 4B, the activated NIR‐II fluorescence of GAP9 distinctly delineated the tumor, which was subsequently excised from a mouse to simulate conventional clinical surgery. Although the primary tumor mass was largely removed during the initial surgical procedure, some residual tumor margin tissues remained, as evidenced by the distinct NIR‐II fluorescence signals in areas indicated by red arrows (Figure 4B and Movie S1). The surgical margins were widened until no fluorescent signals were detected. The tissues resected during both surgical procedures were further validated by H&E staining. As depicted in Figure 4C, GAP9‐guided second surgery consistently resulted in negative surgical margins, indicating the effectiveness of GAP9 in intraoperative monitoring of surgical margins.

To further assess the efficacy of GAP9 in facilitating the resection of residual tumor foci, GAP9 was intravenously injected into 4T1 tumor‐bearing mice 6 h prior to surgery. After initial tumor resection under the guidance of GAP9 fluorescence, faint fluorescence signals were still observed at the surgical site, indicating the presence of residual tumors located deeper within the tissues (Figure 4D and Movie S2). Subsequently, a second resection was conducted under fluorescence guidance to ensure the complete removal of the residual tumor. H&E staining further confirmed that the signal was highly tumor‐specific, and even ultrasmall foci ≤1 mm that are invisible to the naked eye could be detected and completely excised, validating the reliability of GAP9 for the specific identification and removal of tumor tissue (Figure 4E).

### 2.5 GAP9‐Enabled Accurate Detection of Metastatic Tumors

Having demonstrated the capability of GAP9 to illuminate the subcutaneous 4T1 tumor intraoperatively, we proceeded to evaluate its effectiveness in detecting tumor lesions in the peritoneal carcinomatosis mouse model by the inoculation of luciferase‐expressed 4T1 cells directly into the peritoneal cavity. As shown in Figure 4F, bioluminescence imaging (BLI) revealed four metastatic sites. However, due to the limited sensitivity of NIR‐I imaging, GAP9 detected only three metastatic foci within the NIR‐I imaging window. Conversely, all metastatic lesions were successfully identified within the NIR‐II window, consistent with the results of H&E staining and thereby demonstrating the accuracy of GAP9 in detecting metastatic lesions. In an alternative peritoneal metastasis mouse model, the metastatic lesions coalesced into a confluent mass, complicating the identification of individual foci (Figure 4G). BLI was able to distinguish only two metastatic sites that were more widely separated due to its shorter wavelength and limited penetration depth, and resolution. Within the NIR‐I imaging window, four metastatic sites are barely distinguishable. In contrast, NIR‐II imaging enabled by GAP9's enhanced resolution successfully identified all six metastatic sites, which were further confirmed by H&E staining results.

**FIGURE 5:**
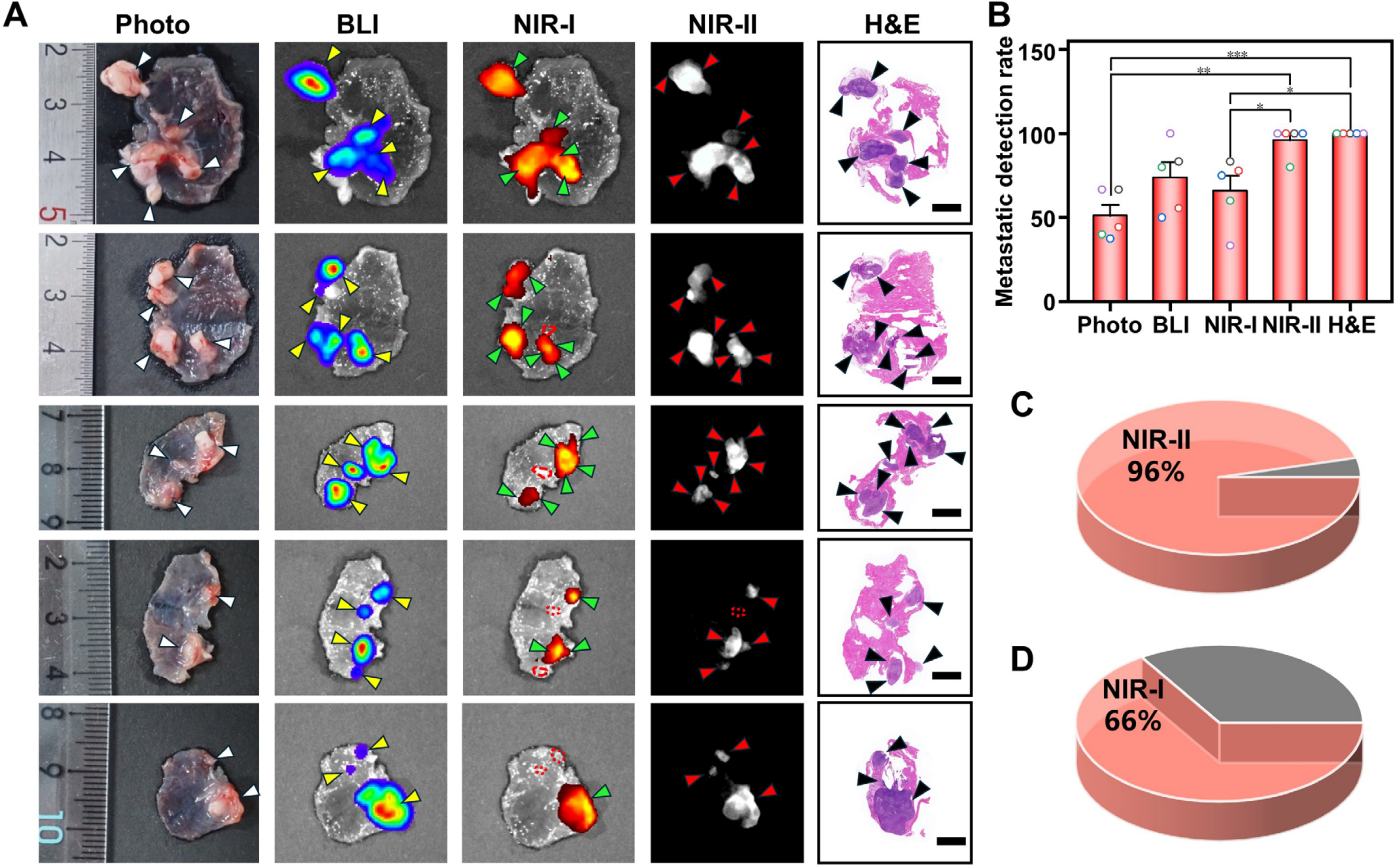
Detection performance of GAP9 for peritoneal metastatic tumors in mice. (A) Multimodal comparison (Photo, BLI, NIR‐I, NIR‐II, and H&E staining) for detecting peritoneal metastatic tumors after intravenous injection of GAP9 (n=5). Scale bars: 5 mm. (B) Statistical comparison of detection rates for peritoneal metastatic tumors using H&E staining, NIR‐II, NIR‐I, BLI, and white‐light photography. Data were expressed as mean ± SD, n = 5. Statistical analysis was performed using one‐way ANOVA with a Tukey's post hoc test. ^*^ *p* < 0.05, ^**^ *p* < 0.01, ^***^ *p* < 0.001. (C)‐(D) Detection rates of NIR‐II (C) and NIR‐I (D) imaging for peritoneal metastatic tumors, with H&E staining counts as the gold standard.

The exceptional tumor specificity of GAP9 inspired us to investigate its potential for accurate intraoperative imaging and image‐guided tumor resection in the case of abdominal metastasis. To this end, we conducted a comprehensive evaluation of metastatic tumor detection in five groups of mice bearing peritoneal metastasis, using different modalities: white light, BLI, NIR‐I fluorescence imaging, NIR‐II fluorescence imaging, and H&E staining. The accuracy of metastatic tumor detection was determined based on H&E staining results, which served as the gold standard. As shown in Figure 5A, only larger metastatic tumors were visible under white light, leading to the detection of a limited number of tumors. Although BLI operates at a shorter wavelength than NIR‐I imaging, its intrinsic bioluminescent properties facilitated the detection of a greater number of metastatic tumors compared to NIR‐I imaging. However, due to limitations in resolution, numerous adherent metastatic lesions remained undetectable. In contrast, NIR‐II imaging exhibited superior efficacy in detecting metastatic tumors, successfully identifying nearly all metastatic sites, with results highly consistent with those from H&E staining. Quantitative analysis provided additional confirmation of this observation (Figure 5B). Notably, the metastatic tumor detection rate utilizing NIR‐II imaging achieved 96% (Figure 5C), in comparison to 66% with NIR‐I imaging (Figure 5D). These results validated the reliability of GAP9 as a robust tool for detecting metastatic tumors during surgical procedures.

## 3 Conclusion

In summary, this study introduced a redox‐capacity engineering strategy for the development of GAP9, a GSH‐activatable MRI/NIR‐II probe designed for precise tumor imaging and image‐guided surgery. Unlike conventional methods that employ rigid probe activation thresholds, we meticulously adjusted the redox capacity of the GAPs nanostructures via pH‐controlled synthesis. This adjustment enabled the activation window of GAP9 to precisely match elevated GSH levels in tumors (0.5–10 mm). This precise redox‐matching strategy ensured that GAP9 remained inactive in normal tissues while undergoing rapid and specific activation at tumor sites, resulting in a 70‐fold increase in signal intensity. Thanks to its specific responsiveness to GSH, GAP9 effectively minimized false‐positive signals from healthy organs and false‐negative detections of microtumor foci with a high T/N ratio of 12, thereby achieving high‐fidelity preoperative tumor localization using MRI and real‐time NIR‐II fluorescence‐guided surgery, with histologically confirmed negative margins. Notably, the specificity provided by this redox engineering strategy enabled GAP9 to detect submillimeter lesions and micrometastases, achieving a 96% detection rate in a mouse model of peritoneal metastasis. This study highlights the potential of rational redox engineering in the development of next‐generation activatable probes. GAP9 exemplifies a high‐precision theranostic platform t with the potential to significantly improve both preoperative staging and intraoperative decision‐making in the field of oncology.

## 4 Experimental Section

### 4.1 Synthesis of IR820‐NH_2_

IR820 (82.6 mg, 0.1 mmol), 4‐Aminothiophenol (25.0 mg, 0.12 mmol), and K_2_CO_3_ (26.5 mg, 0.24 mmol) were dissolved in dry DMF (5 mL) and reacted at room temperature for 24 h. The reaction was monitored by HPLC. Upon completion, the product was purified by HPLC and freeze‐dried to obtain IR820‐NH_2_ with a yield of 81.2%. ^1^H NMR (600 MHz, DMSO‐*d_6_*) δ 8.87 (d, *J* = 14.3 Hz, 2H), 8.27 (d, *J* = 8.6 Hz, 2H), 8.05 (t, *J* = 8.6 Hz, 4H), 7.77 (d, *J* = 9.0 Hz, 2H), 7.62 (t, *J* = 7.6 Hz, 2H), 7.49 (t, *J* = 7.9 Hz, 2H), 7.10 (d, *J* = 8.7 Hz, 2H), 6.57 (d, *J* = 8.8 Hz, 2H), 6.38 (d, *J* = 14.4 Hz, 2H), 5.15 (s, 2H), 4.30 (t, *J* = 7.7 Hz, 4H), 2.76 (t, *J* = 6.4 Hz, 4H), 2.52 (d, *J* = 7.4 Hz, 2H), 1.91 (t, *J* = 5.9 Hz, 2H), 1.86 (d, *J* = 8.4 Hz, 3H), 1.84 (s, 12H), 1.80 – 1.73 (m, 5H). ^13^C NMR (600 MHz, DMSO‐*d_6_*) δ 173.32, 152.59, 148.19, 145.14, 140.31, 133.92, 133.72, 131.79, 130.78, 130.33, 129.13, 128.09, 127.97, 125.25, 122.76, 121.42, 115.53, 112.27, 101.64, 51.20, 50.95, 44.21, 27.43, 26.83, 26.35, 23.02, 21.15. ESI‐MS(m/z): [M^–^] calcd: 914.3337, found 914.3331.

### 4.2 Synthesis of I PAA‐R820

Take a certain amount of polyacrylic acid solution (30% solid content) and the prepared IR820‐NH_2_, along with NHS and EDC, and dissolve them in 100 µL of anhydrous DMSO. After shaking at room temperature for 24 h, the solution turns purple‐red.

### 4.3 Synthesis of GAP

Prepare a sodium hydroxide solution of a certain concentration, take 155 µL, and add it to a 10 mL round‐bottom flask containing deionized water. Slowly add the previously obtained purple‐red product into the solution, stir well, and the solution will turn burgundy. An aqueous manganese chloride solution of specified concentration was prepared. Under ultrasonic agitation, 200 µL aliquots were taken and added dropwise to the aforementioned solution. After the addition was complete, the solution will appear as a dark red emulsion and should be continuously stirred on a magnetic stirrer while avoiding light. Measure 30 mL of isopropanol and slowly add it along the wall of the beaker into the above solution. After stirring for 30 min, add the prepared sodium hydroxide solution dropwise until the solution gradually turns brown. (In this step, the valence states of manganese ions were regulated through the dropwise addition of varying amounts of sodium hydroxide or the introduction of H_2_O_2_, thereby adjusting their GSH‐responsive range.) Allow the reaction to proceed overnight (approximately 12 h), then centrifuge to separate, wash three times with deionized water, and discard the supernatant after the final centrifugation. Add 1 mL of MES buffer with a pH of 8.5 and sonicate for dispersion to perform PEG surface modification. The solution was then added dropwise to pre‐synthesized nanoparticles under continuous stirring and allowed to react for 12 h. After the reaction, the nanoparticles were collected by centrifugation, washed three times with deionized water to remove unbound reagents, and redispersed in 1 mL of phosphate‐buffered saline (PBS). The final nanoparticle suspension was stored in the dark at 4°C for subsequent use.

### 4.4 Cell imaging

4T1, MCF‐7, and Hep G2 cells were seeded in 6‐well plates (approximately 5 × 10^5^ cells per well) and cultured overnight. When the cells reached 80–90% confluence, they were washed with PBS. The cells were then incubated with GAP9 for 3 h, with triplicate samples prepared for each cell line. After incubation, the cells were washed three times with PBS (pH 7.4) to minimize background interference. Prior to probe incubation, cells were pretreated with N‐ethylmaleimide (a thiol‐blocking agent, 1 mm) for 30 min. Control groups for both 4T1, MCF‐7, and Hep G2 cells were treated with equal volumes of PBS. Cell imaging was performed using an NIR‐II in vivo imaging system.

### 4.5 Animal Models Construction

Animal protocols related to this study were reviewed and approved by the Institutional Animal Care and Use Committee of the Fourth Military Medical University (approval number: 20 220 310). All applicable institutional guidelines for the care and use of animals were followed. BALB/c nude female mice were supplied by the Animal Center of the Fourth Military Medical University (FMMU) and used at 6–7 weeks of age. A subcutaneous tumor model was established by injecting 4T1 cells (5 × 10^4^ per mouse) into the right hip. One week after inoculation, animals with tumor size 100–200 mm^3^ were used for imaging studies. For the peritoneal carcinomatosis‐bearing mouse model, luciferase‐expressed 4T1 cells suspended in 1× PBS (5 × 10^4^ per mouse) were intraperitoneally injected into the Female BALB/c nude mice. Mice were monitored by bioluminescence imaging upon injection of a solution of D‐luciferin (150 mg kg^−1^), and small tumor nodules were formed and spread in the peritoneal cavity of mice between 5–7 days after seeding 4T1 cells.

### 4.6 In Vivo Fluorescence Imaging of Tumors

Tumor‐bearing mice were divided into groups (n = 5) based on tumor volume and intravenously injected via the tail vein with the probe dissolved in PBS (pH 7.4, 2.0 mg kg^−1^ per mouse). Fluorescence signals were monitored at various time points post‐injection using an IVIS Spectrum imaging system and a Series II 900/1700‐H imaging system. Major organs (heart, liver, spleen, lungs, kidneys, stomach, and intestines) and tumors were excised for ex vivo imaging analysis. Fluorescence intensities of tumors and organs were normalized in all experimental groups. Peritoneal metastasis was observed 12 h post‐injection. For intraoperative imaging, anesthetized mice underwent laparotomy, followed by bioluminescent and near‐infrared (NIR‐I/NIR‐II) fluorescence imaging. Imaging Parameters: IVIS Spectrum: excitation wavelength = 790 nm, emission wavelength = 845 nm. Series II 900/1700‐H: 808 nm laser excitation, LP1000 filter, laser power = 3 W, exposure time = 50 ms. Region‐of‐interest (ROI) analysis was performed to quantify the tumor‐to‐normal tissue ratio.

### 4.7 In Vivo MRI of Tumors

For T1‐weighted MR imaging, GAP9 (2 mg kg^−1^) was administered intravenously into tumor‐bearing mice (n = 5 for each group). T1‐weighted MR images in the tumors were recorded before and at 2, 4, 6, 8, and 12 h after administration using a 3.0 T clinical MRI instrument. T1‐weighted MR imaging of tumor sections was performed with a fast spin echo sequence: TR = 300 ms, TE = 5.13 ms, slice thickness = 1 mm, FoV = 30 × 30.

### 4.8 Statistical Analysis

Data were presented as the mean ± standard deviation (SD) from at least three independent experiments. The sample size (n) for each experiment was indicated in the corresponding figure legends. For comparisons involving three or more groups, one‐way analysis of variance (ANOVA) followed by Tukey's post hoc test was applied. ^*^: *P* < 0.05 was considered statistically significant. ^**^: *P* < 0.01 and ^***^: *P* < 0.001 was considered highly statistically significant. All statistical analyses were conducted using Origin 2021.

## Conflicts of Interest

The authors declare no conflicts of interest.

## Supporting information




**Supporting File 1**: advs73671‐sup‐0001‐SuppMat.docx.

**Supporting File 2**: advs73671‐sup‐0002‐Movie 1.avi.

**Supporting File 3**: advs73671‐sup‐0003‐Movie 2.avi.

## Data Availability

The data that support the findings of this study are available from the corresponding author upon reasonable request.
