# Supplementary material for: Redox‐Programmed Activation of a Dual‐Modal Probe for High‐Fidelity Tumor Delineation and Image‐Guided Surgery
Source: Adv Sci (Weinh). 2026 Jan 4;13(16):e19223. doi: 10.1002/advs.202519223 (PMC13042449; doi:10.1002/advs.202519223)
Supplement: Supplementary file 1 — Supporting File 1: advs73671‐sup‐0001‐SuppMat.docx. [file ADVS-13-e19223-s001.docx]

**Redox-Programmed Activation of a Dual-Modal Probe for High-Fidelity Tumor Delineation and Image-Guided Surgery**

Haohao Yan^‡^, Yanbin Feng^‡^, Qian Jia*, Ruili Zhang*, Zhongliang Wang*

**1. Experimental Section Supporting Information**

**1.1 Materials**

All chemical used in the experiments were purchased from Sigma-Aldrich and Alfa Aesar unless otherwise stated. Commercially available reagents were used directly without further purification, unless otherwise indicated. Polyacrylic acid (PAA, Mw: 3000), methoxy polyethylene glycol amine (mPEG-NH₂, Mw: 3000), and isopropyl alcohol (IPA) were obtained from Macklin Inc. (Shanghai, China). Deionized water (DI water) was used in all experiments. The murine breast cancer cell line 4T1, human hepatocellular carcinomas cell line Hep G2 and human breast cancer cell line MCF-7 were procured from Shanghai Zhong Qiao Xin Zhou Biotechnology Co., Ltd. (Shanghai, China). All cell lines were tested for mycoplasma contamination prior to use. Penicillin-Streptomycin, Dulbecco’s Modified Eagle Medium (DMEM), RPMI 1640 medium, fetal bovine serum (FBS), and 0.25% (w/v) trypsin solution were purchased from Gibco Life Technologies (AG, Switzerland). Phosphate-buffered saline (PBS) was sourced from OpticsPlanet, Inc. (USA). Female BALB/c nude mice (6–7 weeks old, 18–22 g) were provided by the Animal Center of the Fourth Military Medical University (Xi’an, China).

**1.2 Characterization**

High-performance liquid chromatography (HPLC) analyses were performed on a xgt6515-10s system using a methanol (MeOH)/water mixture as the eluent. Transmission electron microscopy (TEM) images were obtained using the JEM-2100 F electron microscope for direct observation. The hydrodynamic diameter and Zeta potential of samples were performed on a Malvern Zeta Sizer Nano (Malvern Instruments). The crystal structures of the samples were determined by powder X-ray diffraction (PXRD) patterns (Rigaku Ultima IV, Japan). XPS analysis was acquired via an XPS system (AXIS UltraDLD). Absorption spectra were measured on a Shimadzu spectrophotometer (UV-2600) and fluorescence spectra measurements were performed on an Edinburgh FLSP920 fluorescence spectrophotometer. Absorbance in 3-(4,5-dimethylthiazol-2-yl)-2,5diphenyltetrazolium bromide (MTT) assay was measured in a Multiskan (Thermo Scientific). The contents of Mn were analyzed by inductively coupled plasma mass spectrometry (ICP-MS, iCAP RQ, Thermo Fisher). *In vivo* imaging was performed using the NIR-II *in vivo* animal imaging system (Series II 900/1700-H, China), the *in vivo* animal imaging system (IVIS Lumina III, USA). 3T clinical MRI instrument (GE signa 3.0T HD, Milwaukee, WI) was applied *in vivo* T1-weighted MR imaging.

**1.3 Preparation of Probes**

**Synthesis of IR820-NH_2_**

IR820 (82.6 mg, 0.1 mmol), 4-Aminothiophenol (25.0 mg, 0.12 mmol) and K_2_CO_3_ (26.5 mg, 0.24 mmol) were dissolved in dry DMF (5 mL) and reacted at room temperature for 24 h. The reaction was monitored by HPLC. Upon completion, the product was purified by HPLC and freeze-dried to obtain IR820-NH_2_ with a yield of 81.2%. ^1^H NMR (600 MHz, DMSO-*d_6_*) δ 8.87 (d, *J* = 14.3 Hz, 2H), 8.27 (d, *J* = 8.6 Hz, 2H), 8.05 (t, *J* = 8.6 Hz, 4H), 7.77 (d, *J* = 9.0 Hz, 2H), 7.62 (t, *J* = 7.6 Hz, 2H), 7.49 (t, *J* = 7.9 Hz, 2H), 7.10 (d, *J* = 8.7 Hz, 2H), 6.57 (d, *J* = 8.8 Hz, 2H), 6.38 (d, *J* = 14.4 Hz, 2H), 5.15 (s, 2H), 4.30 (t, *J* = 7.7 Hz, 4H), 2.76 (t, *J* = 6.4 Hz, 4H), 2.52 (d, *J* = 7.4 Hz, 2H), 1.91 (t, *J* = 5.9 Hz, 2H), 1.86 (d, *J* = 8.4 Hz, 3H), 1.84 (s, 12H), 1.80 – 1.73 (m, 5H). ^13^C NMR (600 MHz, DMSO-*d_6_*) δ 173.32, 152.59, 148.19, 145.14, 140.31, 133.92, 133.72, 131.79, 130.78, 130.33, 129.13, 128.09, 127.97, 125.25, 122.76, 121.42, 115.53, 112.27, 101.64, 51.20, 50.95, 44.21, 27.43, 26.83, 26.35, 23.02, 21.15. ESI-MS(m/z): [M^–^] calcd: 914.3337, found 914.3331.

**Synthesis of I PAA-R820**

Take a certain amount of polyacrylic acid solution (30% solid content) and the prepared IR820-NH_2_, along with NHS and EDC, and dissolve them in 100 μL of anhydrous DMSO. After shaking at room temperature for 24 hours, the solution turns purple-red.

**Synthesis of GAP**

Prepare a sodium hydroxide solution of a certain concentration, take 155 μL, and add it to a 10 mL round-bottom flask containing deionized water. Slowly add the previously obtained purple-red product into the solution, stir well, and the solution will turn burgundy. An aqueous manganese chloride solution of specified concentration was prepared. Under ultrasonic agitation, 200 μL aliquots were taken and added dropwise to the aforementioned solution. After the addition is complete, the solution will appear as a dark red emulsion and should be continuously stirred on a magnetic stirrer while avoiding light. Measure 30 mL of isopropanol and slowly add it along the wall of the beaker into the above solution. After stirring for 30 minutes, add the prepared sodium hydroxide solution dropwise until the solution gradually turns brown. (In this step, the valence states of manganese ions are regulated through the dropwise addition of varying amounts of sodium hydroxide or the introduction of H_2_O_2_, thereby adjusting their GSH-responsive range.) Allow the reaction to proceed overnight (approximately 12 hours), then centrifuge to separate, wash three times with deionized water, and discard the supernatant after the final centrifugation. Add 1 mL of MES buffer with a pH of 8.5 and sonicate for dispersion to perform PEG surface modification. The solution was then added dropwise to pre-synthesized nanoparticles under continuous stirring and allowed to react for 12 hours. After the reaction, the nanoparticles were collected by centrifugation, washed three times with deionized water to remove unbound reagents, and redispersed in 1 mL of phosphate-buffered saline (PBS). The final nanoparticle suspension was stored in the dark at 4 °C for subsequent use.

**Synthesis of SiO_2_**

In addition, an “always-on” probe was synthesized in this study as a control probe for GAP9 experiments. The synthesis method refers to the work of Sun Meng et al.^[1]^ Briefly, SiO_2_ nanoparticles of approximately 100 nm in size were first synthesized. A mixture of 0.2 g of CTAB, 25 mL of deionized water, 7 mL of methanol, and 50 mL of diethylamine was stirred in a 60 °C water bath for 30 minutes. Then, 2 mL of TEOS was quickly added to the solution. The solution gradually turned white, and after stirring for 2 hours, it was allowed to cool to room temperature. After adding 20 mL of acetone, the mixture was centrifuged. The surface of the collected SiO_2_ particles was aminated and then reacted with PAA connected to IR820 through a condensation reaction. Finally, the particles were centrifuged, washed with deionized water, and collected.

**1.4 Cell lines culture**

Murine mammary carcinoma cell line 4T1, human breast cancer cell line MCF-7, and human hepatocellular carcinomas cell line Hep G2 were cultured in Dulbecco’s modified Eagle’s medium (DMEM) with 10% fetal bovine serum (FBS) at 37 °C under 5% CO_2_.

**1.5 Cytotoxicity test *in vitro***

The cell viability was measured using conventional MTT assay. The used cell lines were human breast cancer cell line MCF-7. Typically, cells were respectively seeded at a density of 5000 cells per well in a 96-well plate. After 12 h of incubation, fresh cell culture medium containing various concentrations (0, 5, 10, 25, 50, 100, and 250 μg mL^−1^) of GAP9 was added for further incubation of 24 h. To determine the cell viability, cells were washed with PBS for three times and incubated with MTT (5 mg mL^−1^) solution for another 4 h, followed by 150 μL of DMSO was added to each and optical absorption at 492 nm was measured to calculate cell viability.

**1.6** **Cell imaging**

4T1, MCF-7, and Hep G2 cells were seeded in 6-well plates (approximately 5×10^5^ cells per well) and cultured overnight. When the cells reached 80–90% confluence, they were washed with PBS. The cells were then incubated with GAP9 for 3 hours, with triplicate samples prepared for each cell line. After incubation, the cells were washed three times with PBS (pH 7.4) to minimize background interference. Prior to probe incubation, cells were pretreated with N-ethylmaleimide (a thiol-blocking agent, 1 mM) for 30 minutes. Control groups for both 4T1, MCF-7 and Hep G2 cells were treated with equal volumes of PBS. Cell imaging was performed using an NIR-II *in vivo* imaging system.

**1.7 Animal models construction**

Animal protocols related to this study were reviewed and approved by the Institutional Animal Care and Use Committee of the Fourth Military Medical University (approval number: 20220310). All applicable institutional guidelines for the care and use of animals were followed. BALB/c nude female mice were supplied by the Animal Center of the Fourth Military Medical University (FMMU) and used at 6–7 weeks of age. A subcutaneous tumor model was established by injecting 4T1 cells (5×10^4^ per mouse) into the right hip. One week after inoculation, animals with tumor size 100–200 mm^3^ were used for imaging studies. For the peritoneal carcinomatosis-bearing mouse model, luciferase-expressed 4T1 cells suspended in 1× PBS (5×10^4^ per mouse) were intraperitoneally injected into the Female BALB/c nude mice. Mice were monitored by bioluminescence imaging upon injection of a solution of D-luciferin (150 mg kg^−1^), and small tumor nodules were formed and spread in the peritoneal cavity of mice between 5-7 days after seeding 4T1 cells.

**1.8 *In vivo* fluorescence imaging of tumors**

Tumor-bearing mice were divided into groups (n = 5) based on tumor volume and intravenously injected via the tail vein with the probe dissolved in PBS (pH 7.4, 2.0 mg kg^−1^ per mouse). Fluorescence signals were monitored at various time points post-injection using an IVIS Spectrum imaging system and a Series II 900/1700-H imaging system. Major organs (heart, liver, spleen, lungs, kidneys, stomach, and intestines) and tumors were excised for *ex vivo* imaging analysis. Fluorescence intensities of tumors and organs were normalized in all experimental groups. Peritoneal metastasis was observed 12 hours post-injection. For intraoperative imaging, anesthetized mice underwent laparotomy, followed by bioluminescent and near-infrared (NIR-I/NIR-II) fluorescence imaging. Imaging Parameters: IVIS Spectrum: excitation wavelength = 790 nm, emission wavelength = 845 nm. Series II 900/1700-H: 808 nm laser excitation, LP1000 filter, laser power = 3 W, exposure time = 50 ms. Region-of-interest (ROI) analysis was performed to quantify the tumor-to-normal tissue ratio.

**1.9 *In vivo* MR imaging of tumors**

For T1-weighted MR imaging, GAP9 (2 mg kg^−1^) were administrated intravenously into tumor-bearing mice (n = 5 for each group). T1-weighted MR images in the tumors were recorded before and at 2, 4, 6, 8, and 12 h after administration using a 3.0 T clinical MRI instrument. T1-weighted MR imaging of tumors sections was performed with a fast spin echo sequence: TR = 300 ms, TE = 5.13 ms, slice thickness = 1 mm, FoV = 30 × 30.

**1.10 Statistical analysis**

Data are presented as the mean ± standard deviation (SD) from at least three independent experiments. The sample size (n) for each experiment is indicated in the corresponding figure legends. For comparisons involving three or more groups, one-way analysis of variance (ANOVA) followed by Tukey’s post hoc test was applied. *: *P* < 0.05 was considered statistically significant. **: *P* < 0.01 and ***: *P* < 0.001 was considered highly statistically significant. All statistical analyses were conducted using Origin 2021.

**2 Supplementary Schemes and Figures**

**
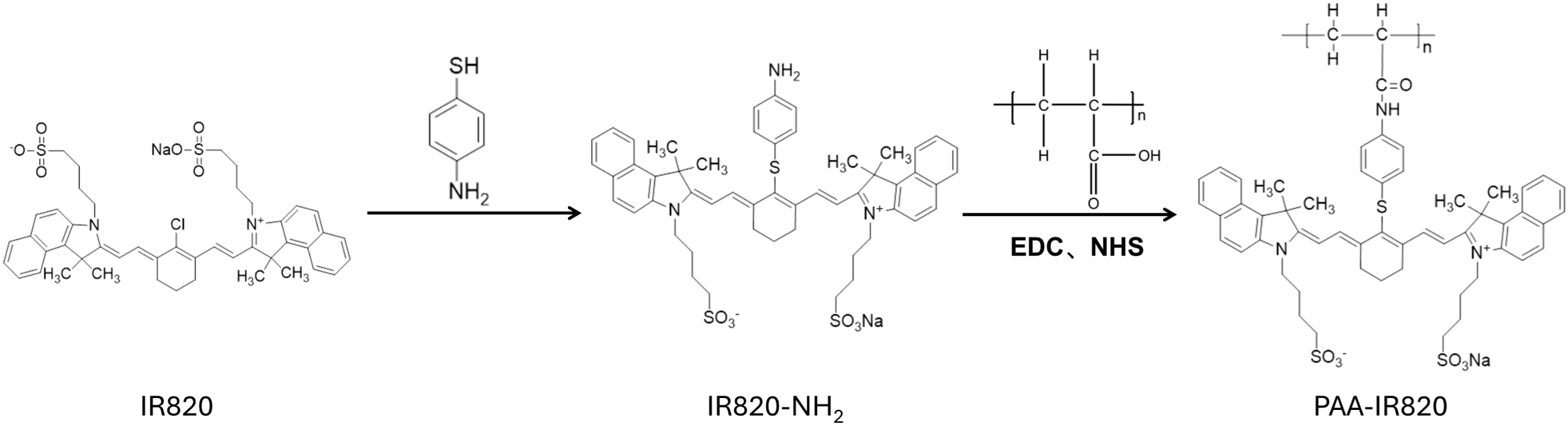
**

**Scheme S1.** Synthesis route of the dyes IR820-NH_2_ and PAA-IR820


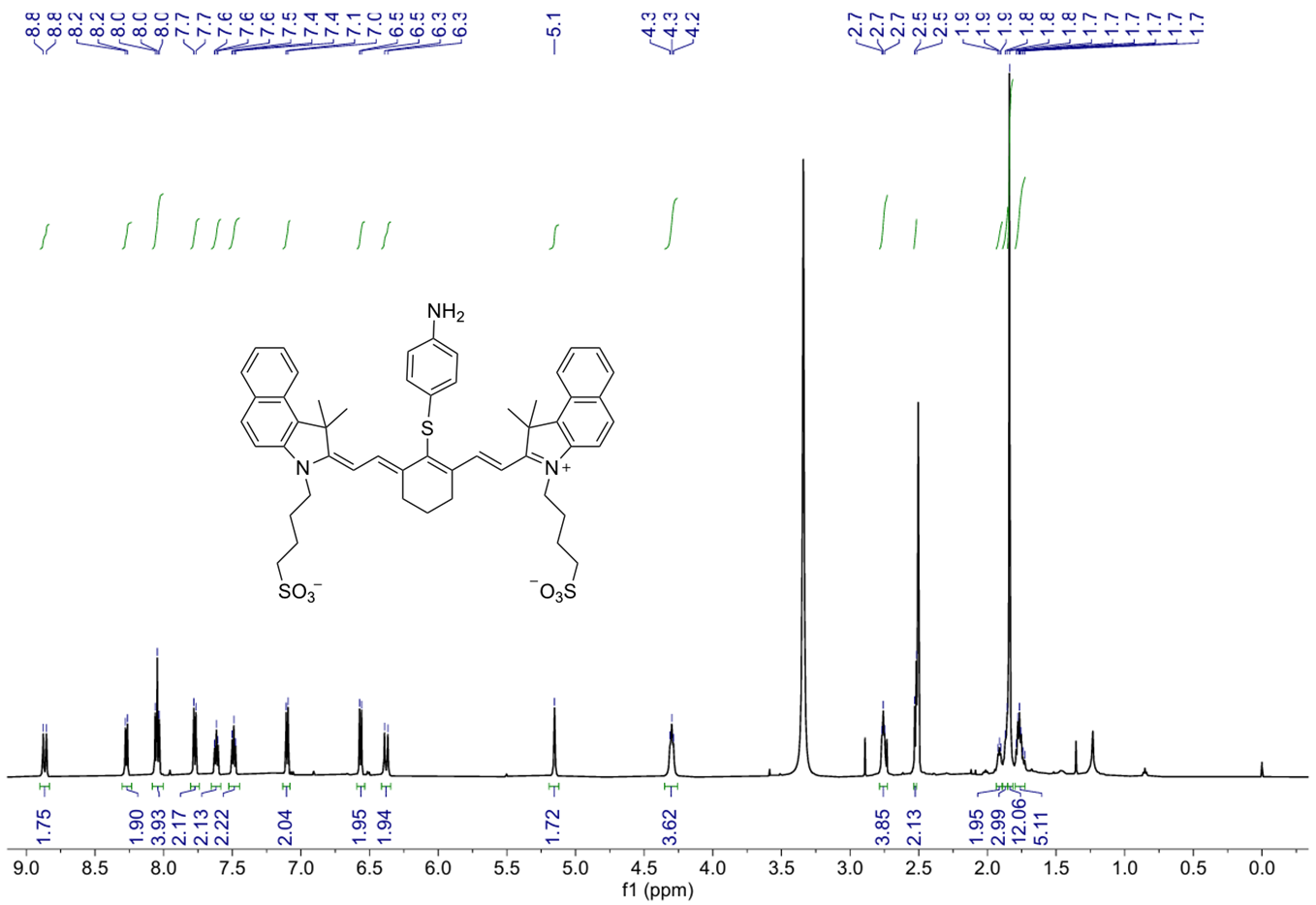


**Figure S1.** ^1^H spectrum of IR820-NH_2_ in DMSO-*d_6_*.


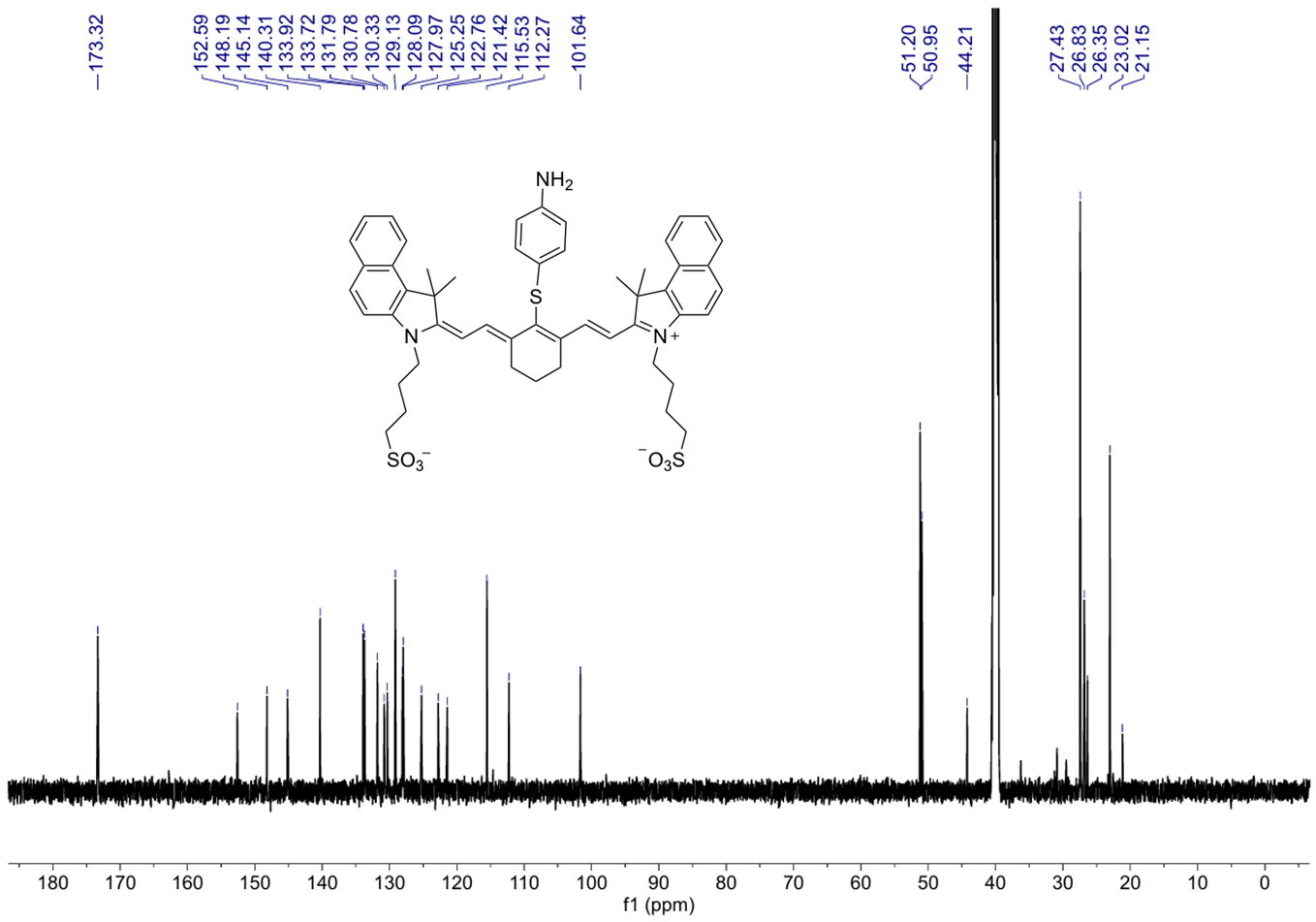


**Figure S2.** ^13^C spectrum of IR820-NH_2_ in DMSO-*d_6_*.


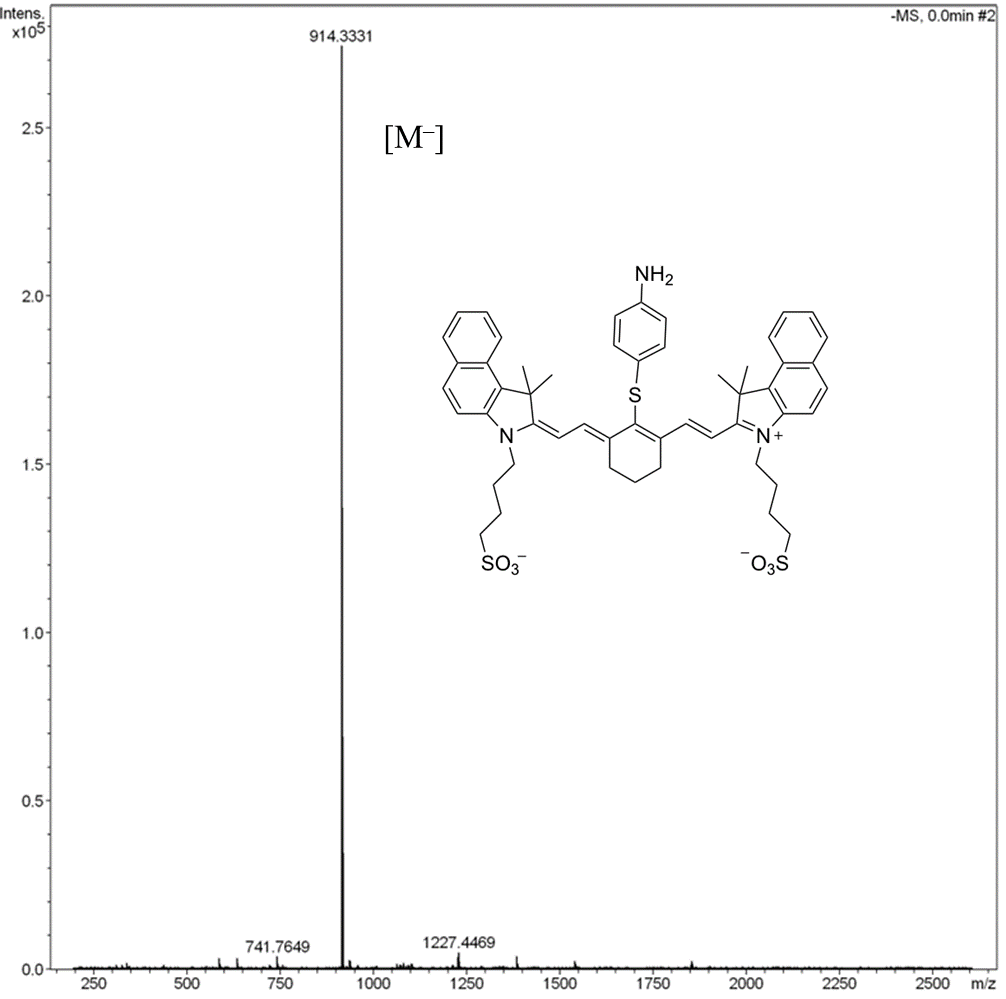


**Figure S3.** ESI-MS of IR820-NH_2_.


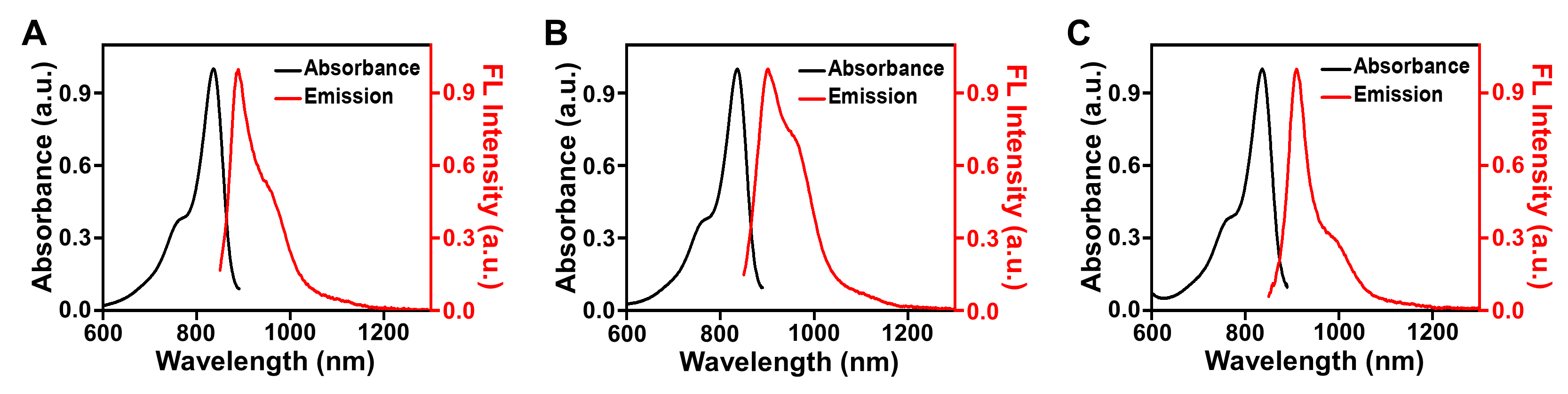


**Figure S4.** Normalized absorption spectra (black line) and emission spectra (red line) of IR820 (A), IR820-NH_2_ (B) and PAA-IR820 (C) excited at 835 nm.


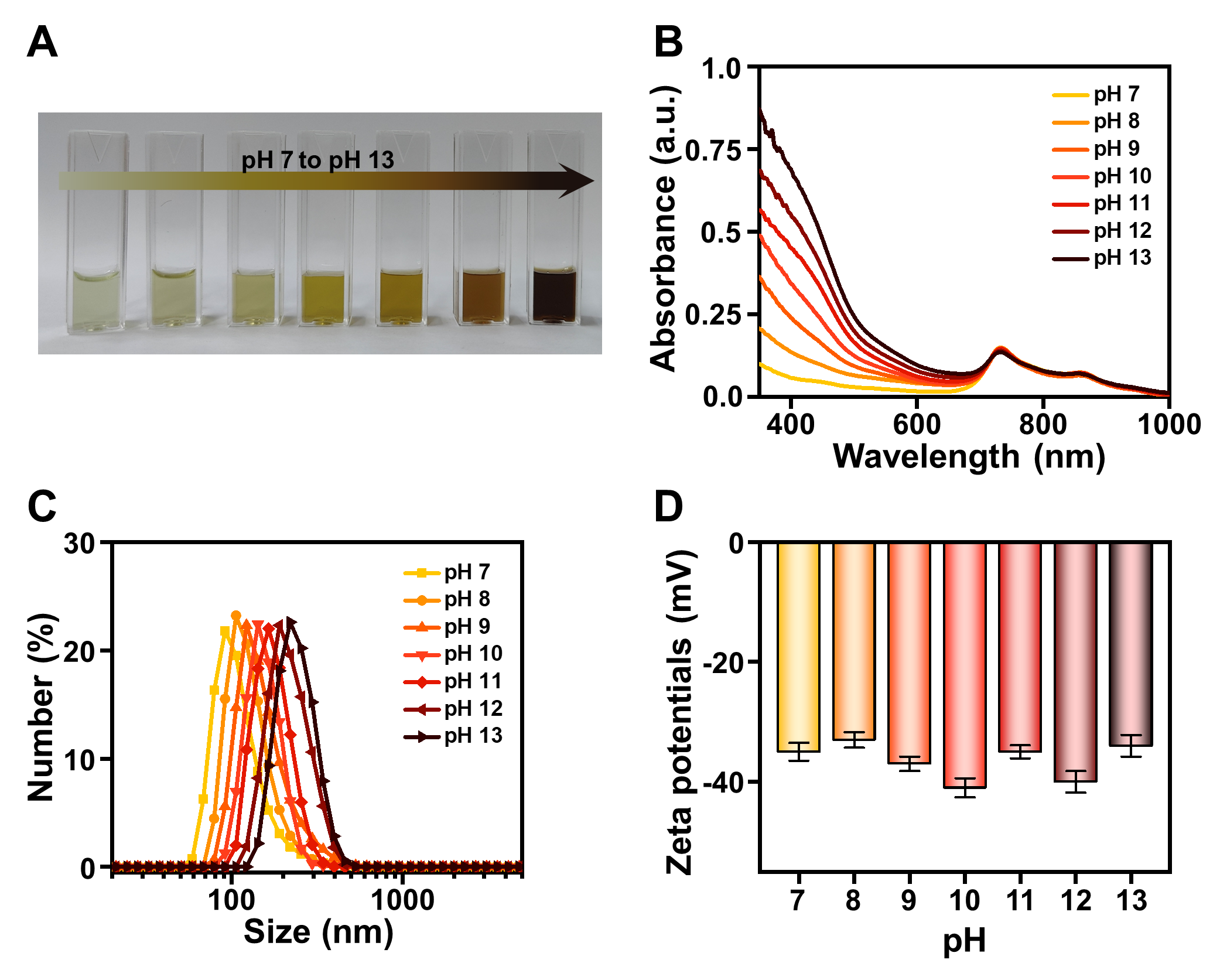


**Figure S5.** Characterization of GAP probes synthesized under different pH conditions. Photographic images (A), Absorption spectra (B), Hydrodynamic diameter (C), and Zeta potential (D) of GAP probes synthesized under pH conditions ranging from 7 to 13. All statistical data are presented as mean values ± SD. (n = 3 independent experiments).


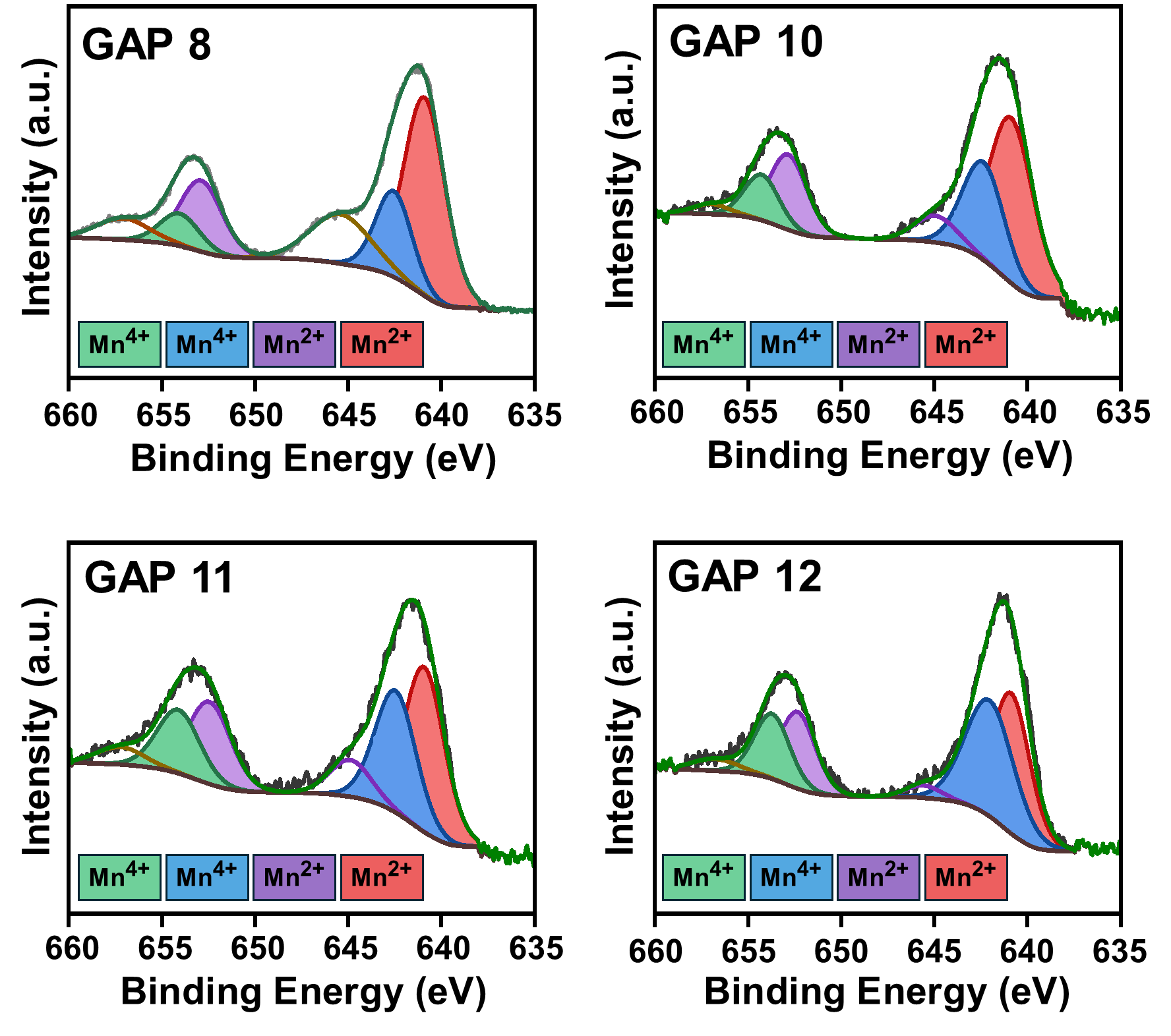


**Figure S6.** Mn 2p XPS spectra of GAP synthesized at pH 8, 10, 11, and 12.


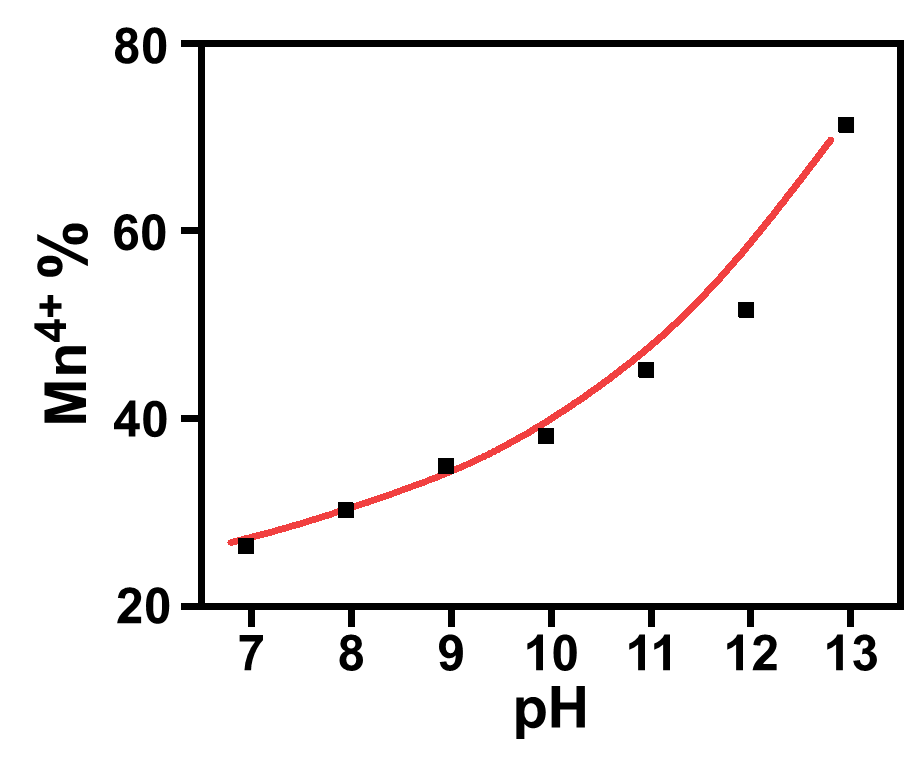


**Figure S7.** pH-dependent Mn^4+^ content variation profile in GAP probes.


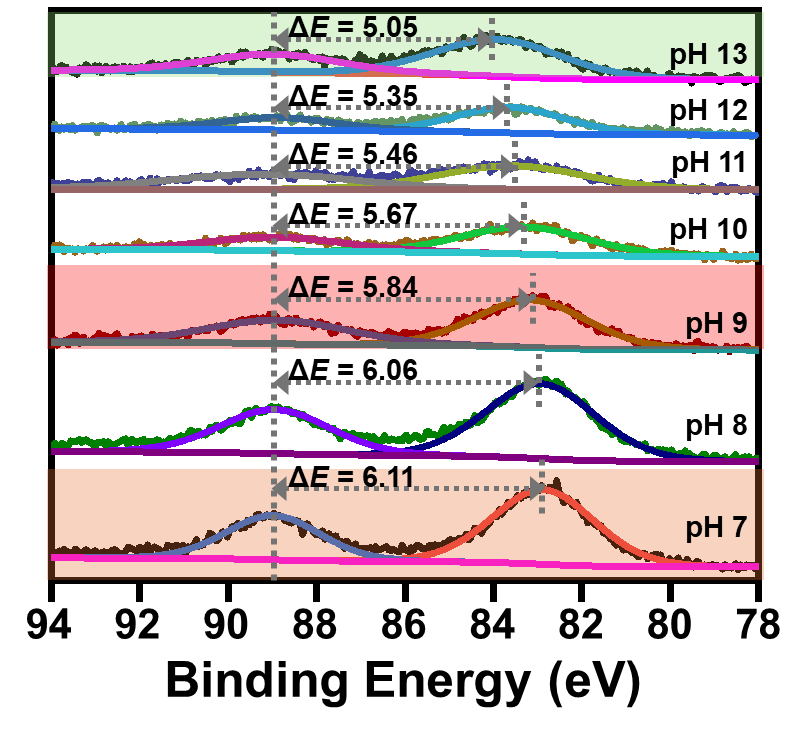


**Figure S8.** Mn 3s XPS spectra of GAP synthesized under different pH conditions.


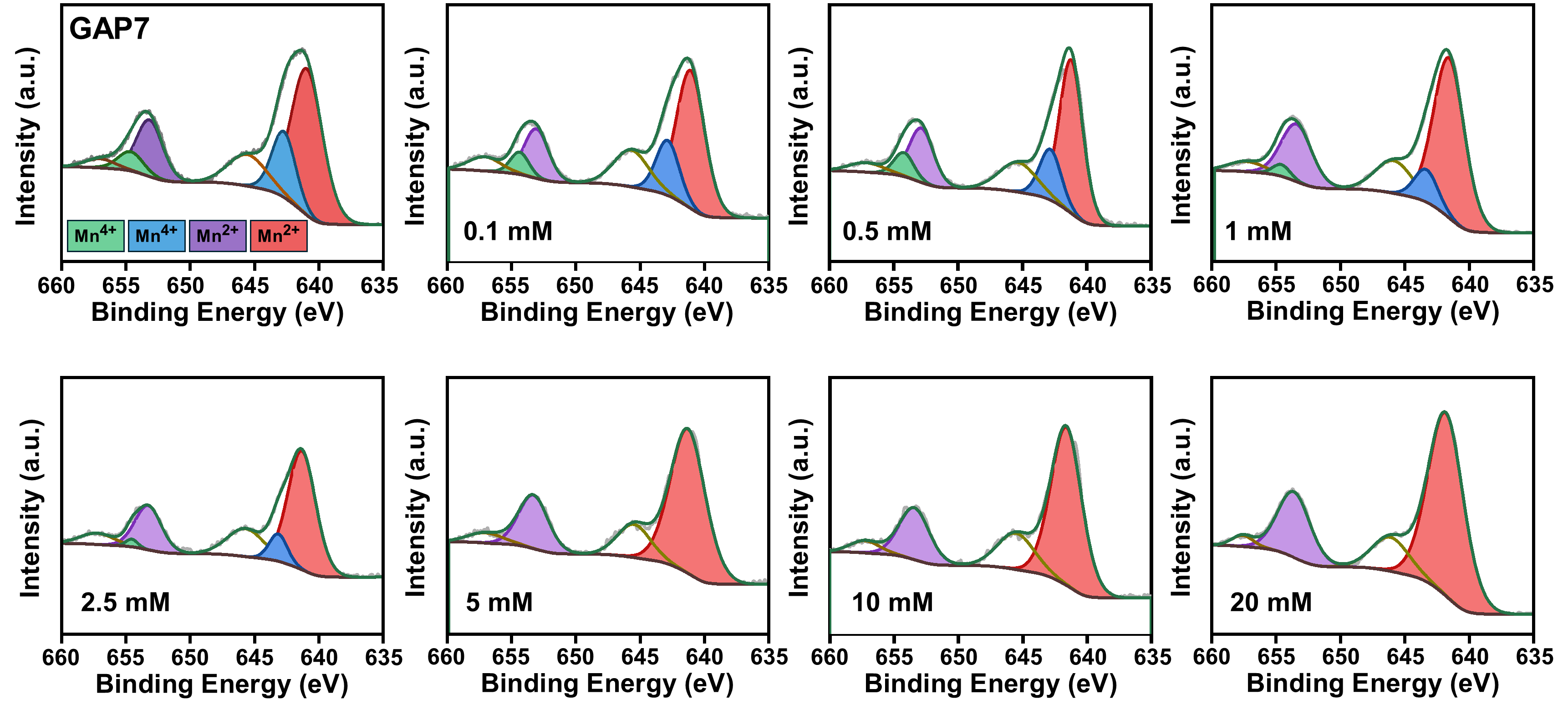


**Figure S9.** GSH concentration-dependent variations in Mn 2p XPS spectra of GAP7.


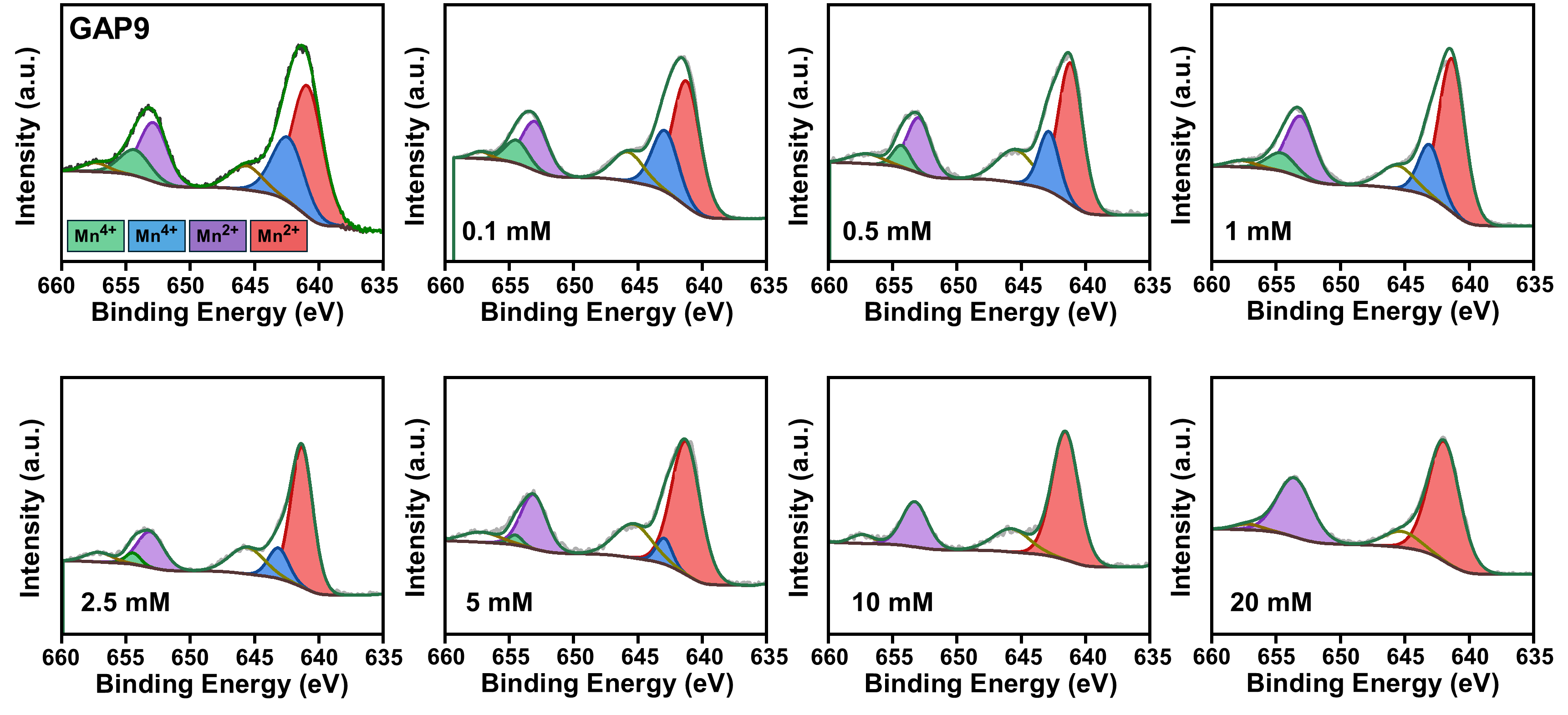


**Figure S10.** GSH concentration-dependent variations in Mn 2p XPS spectra of GAP9.


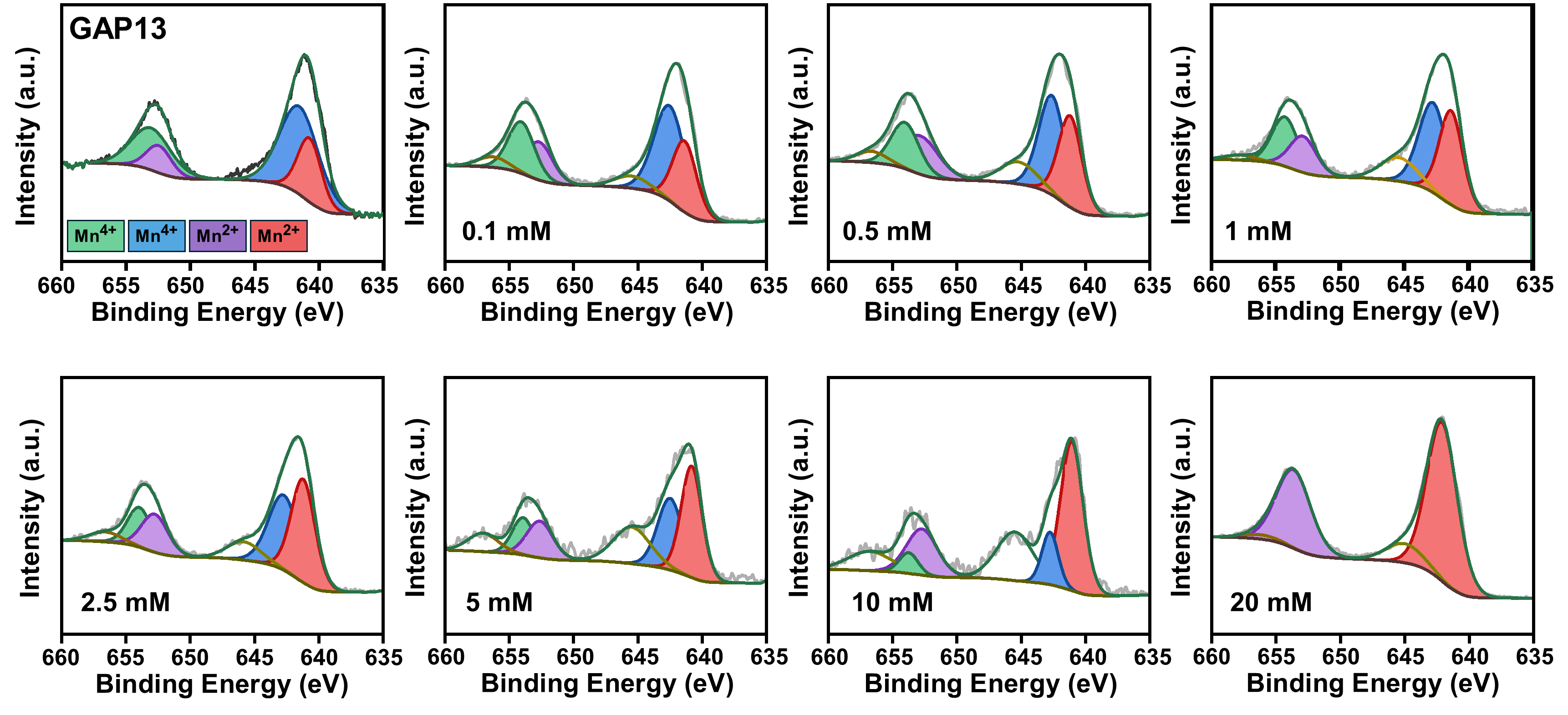


**Figure S11.** GSH concentration-dependent variations in Mn 2p XPS spectra of GAP13.


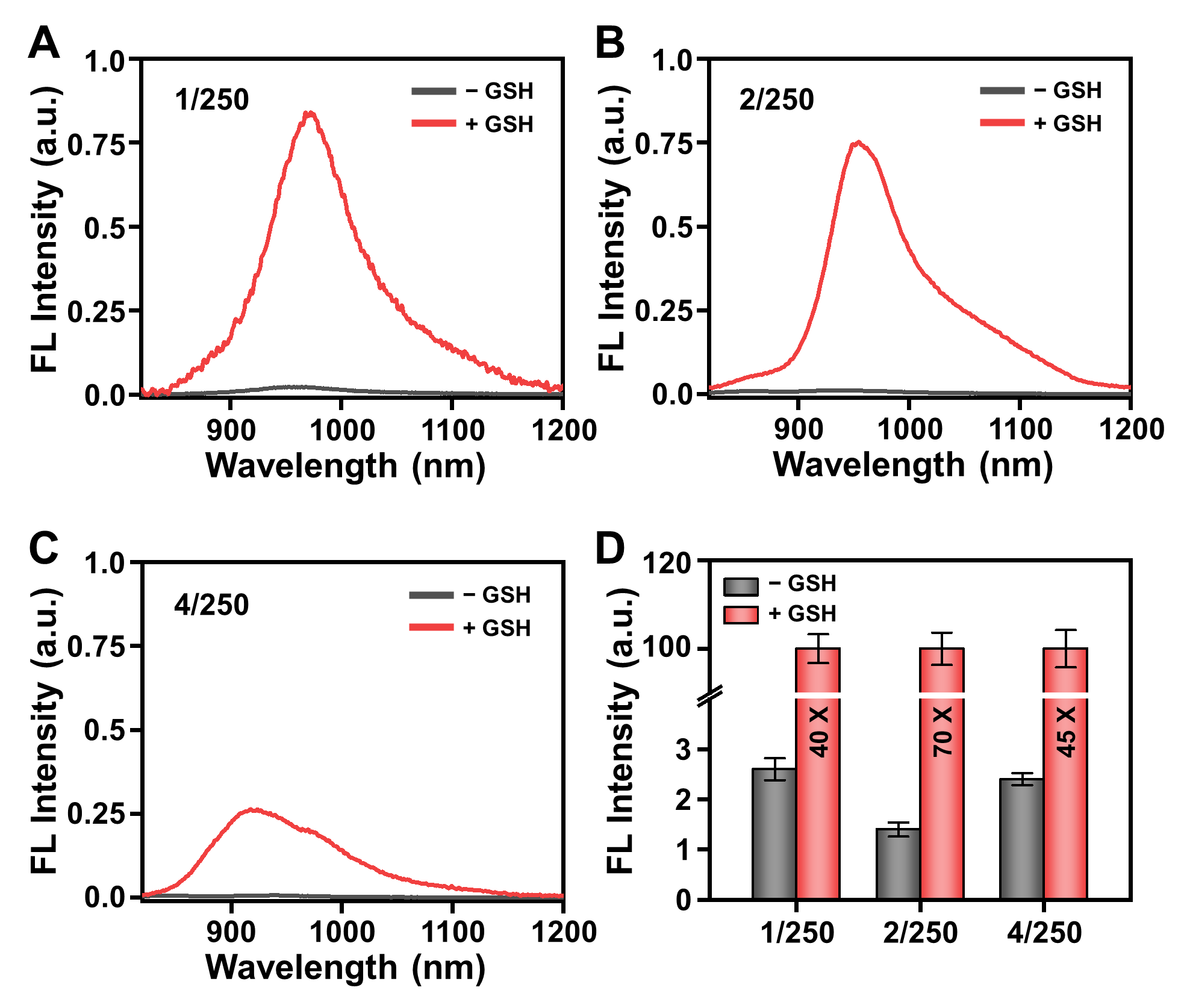


**Figure S12.** Changes in fluorescence spectra of GAP9 with different IR820 conjugation ratios, both in the presence and absence of 10 mM GSH. The amino-to-carboxyl molar ratios are (A) 1/250, (B) 2/250, and (C) 4/250. (D) GSH-dependent normalized fluorescence intensity of GAP9 with different IR820 conjugation ratios. All statistical data are presented as mean values ± SD. (n = 3 independent experiments).


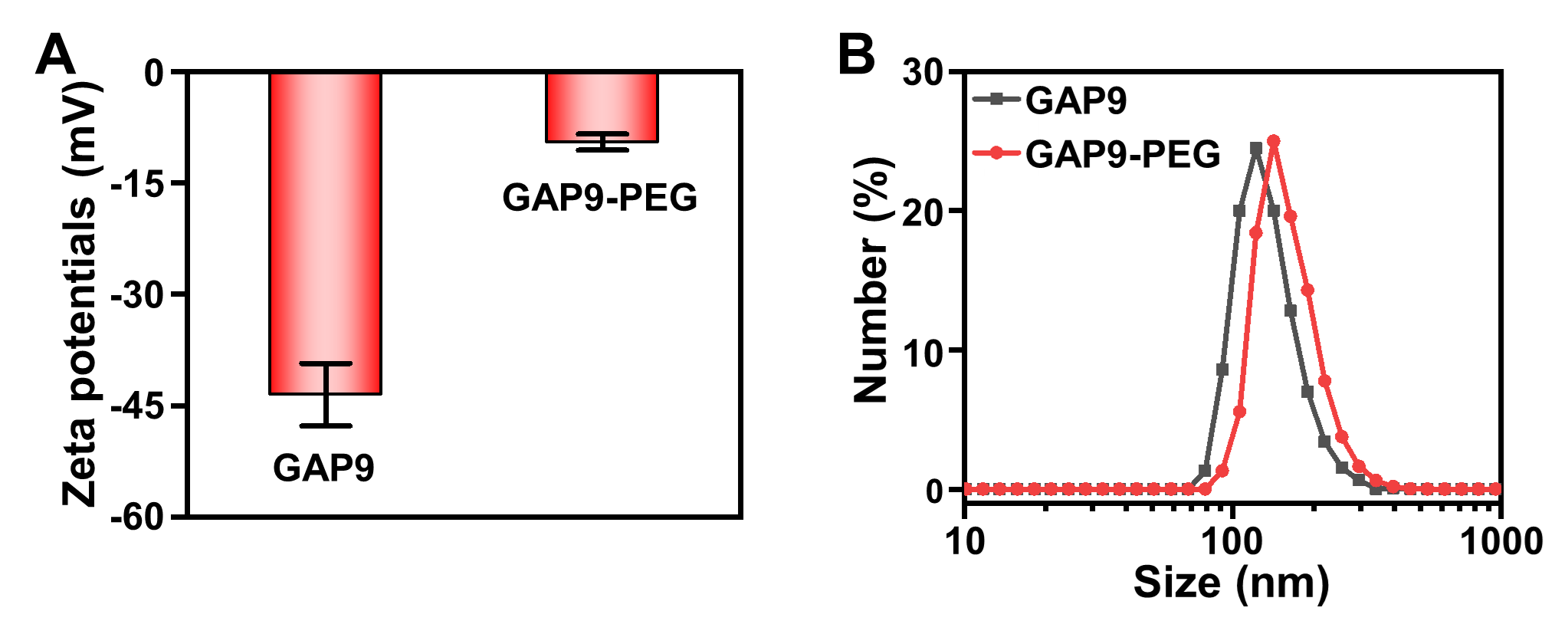


**Figure S13.** (A) Zeta potential and (B) Hydrodynamic diameter of GAP9 before and after PEGylation. All statistical data are presented as mean values ± SD. (n = 3 independent experiments).


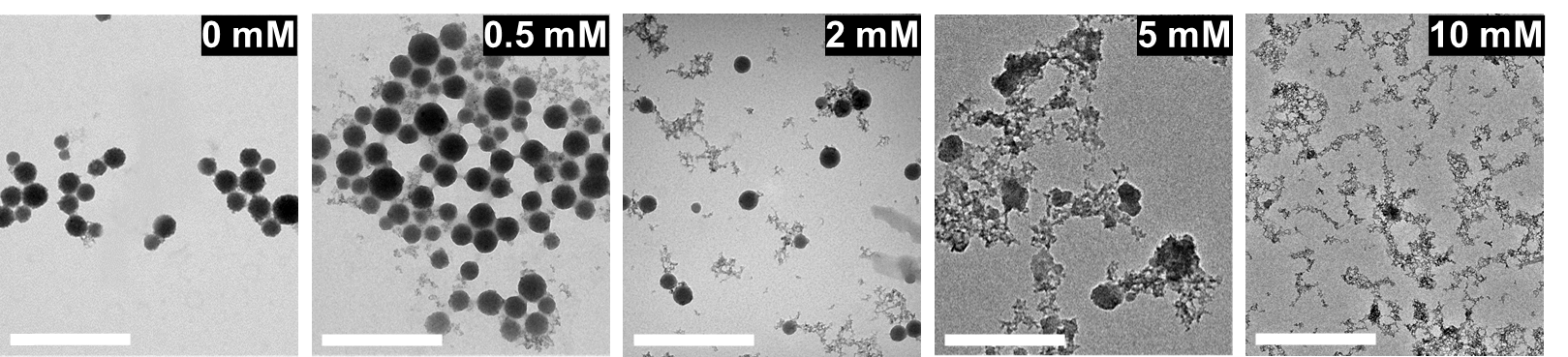


**Figure S14.** TEM images of GAP9 under different GSH concentrations. Scale bar: 500 nm


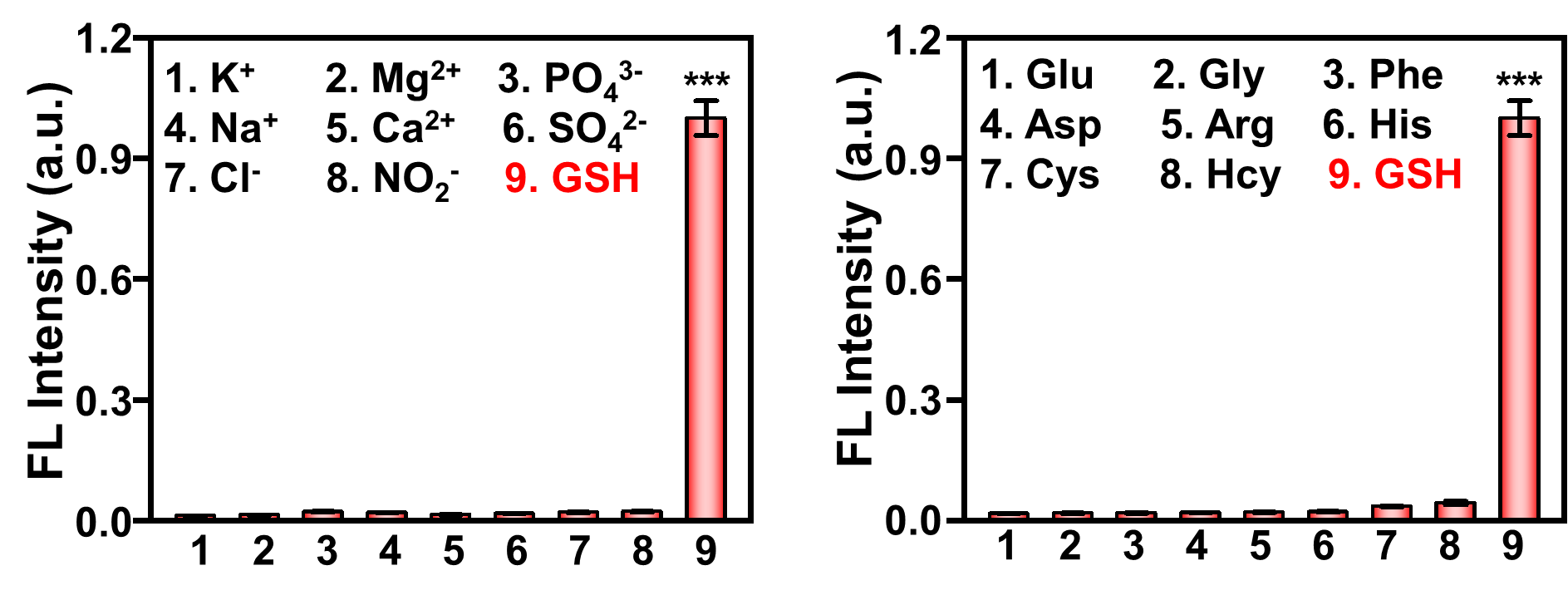


**Figure S15.** The normalized fluorescence intensity of GAP9 at 940 nm in response to various ions, biomolecules. Data were expressed as mean ± SD, n = 5. Statistical analysis was performed using one-way ANOVA with a Tukey’s post hoc test. * *p* < 0.05, ** *p* < 0.01, ***: *P* < 0.001.


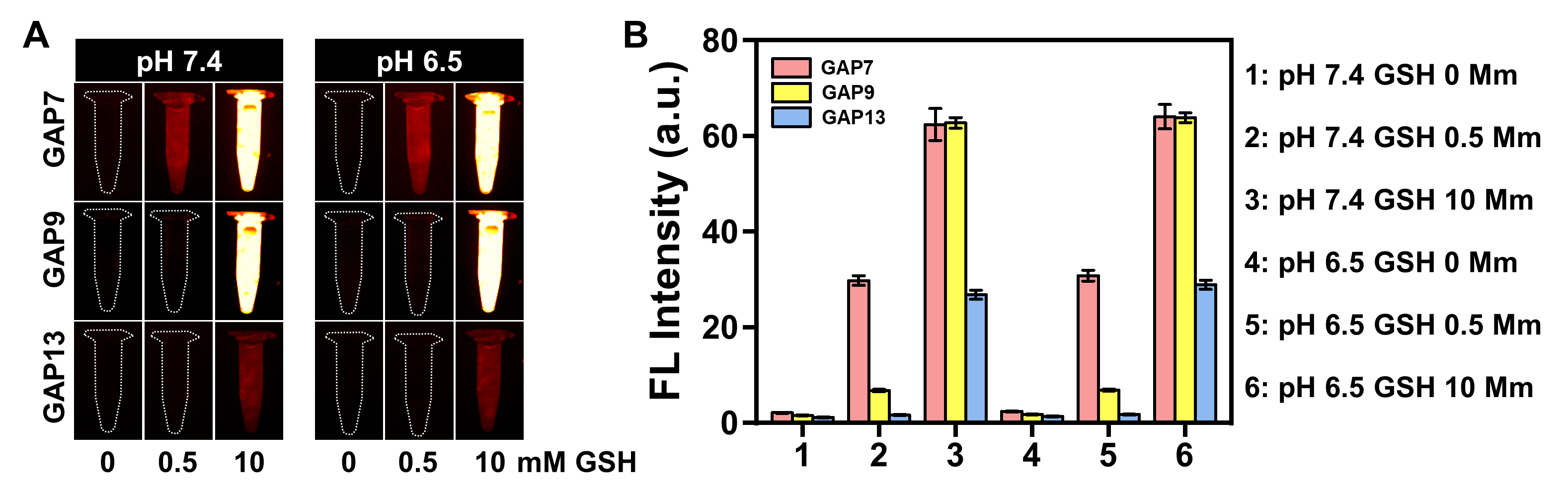


**Figure S16.** NIR-II fluorescence intensity of GAPs in buffers at different pH values (both in the presence and absence of GSH). (B) Quantitative analysis of NIR-II fluorescence intensity from (A). All statistical data are presented as mean values ± SD. (n = 3 independent experiments).


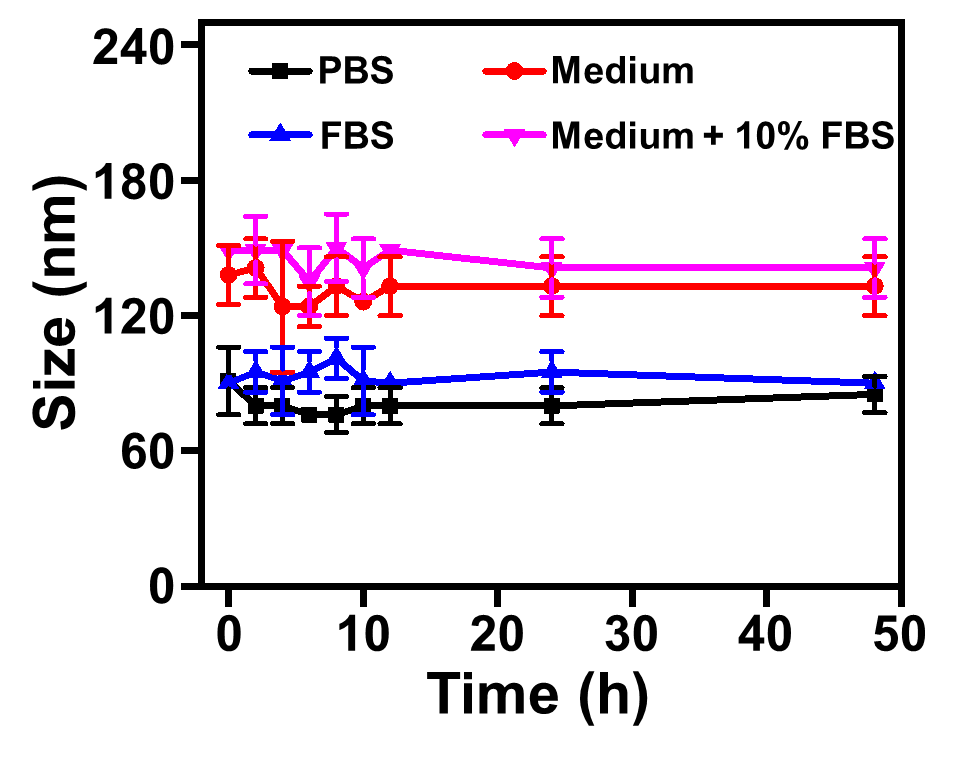


**Figure S17.** Long term dispersity of GAP9 suspended in PBS, medium, FBS, and 10% FBS complete medium, respectively. All statistical data are presented as mean values ± SD. (n = 3 independent experiments).


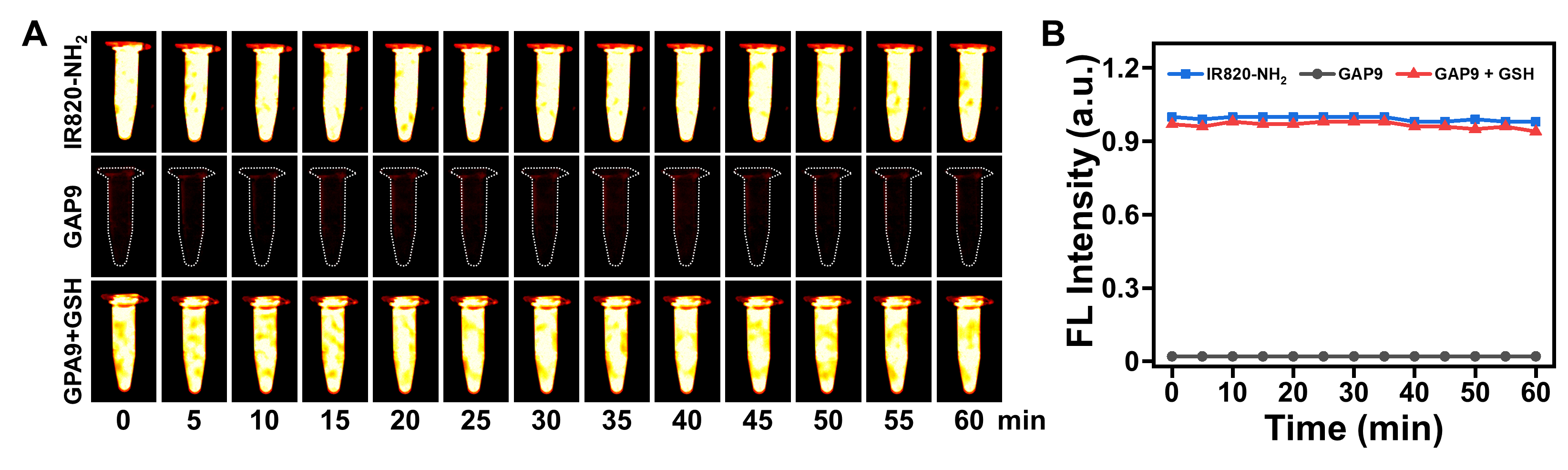


**Figure S18.** Fluorescence stability of IR820 and GAP9 before and after GSH activation under 808 nm laser irradiation (1 W cm^−2^). (B) Quantitative analysis of NIR-II fluorescence intensity from (A).


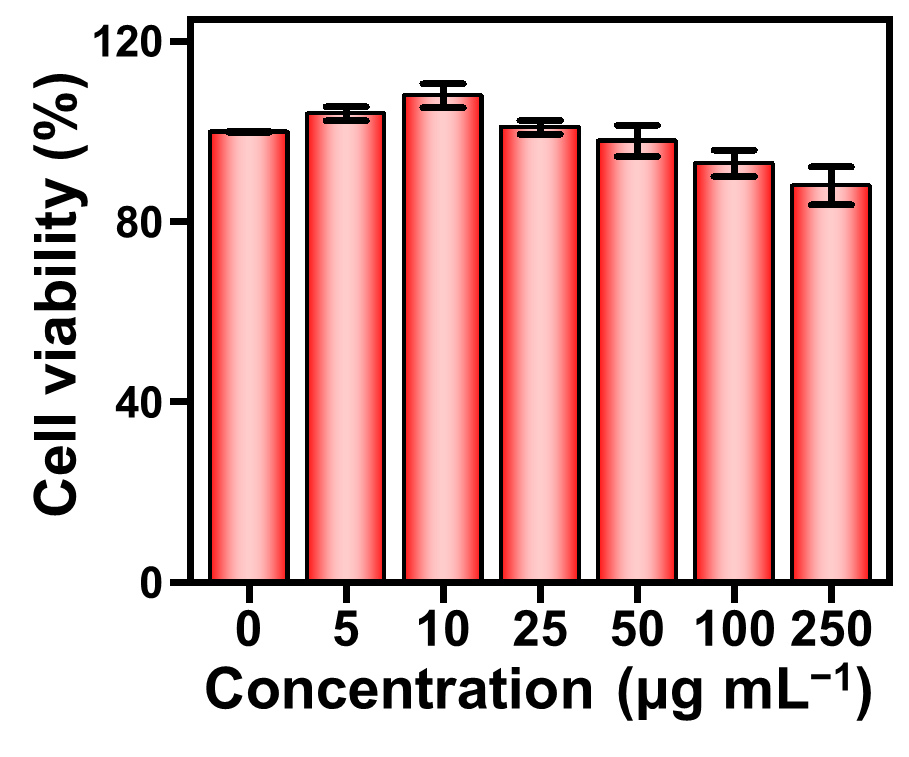


**Figure S19.** Cell viability of 4T1 cells after treatment with different concentrations of GAP9 for 24 h. All statistical data are presented as mean values ± SD. (n = 3 independent experiments).


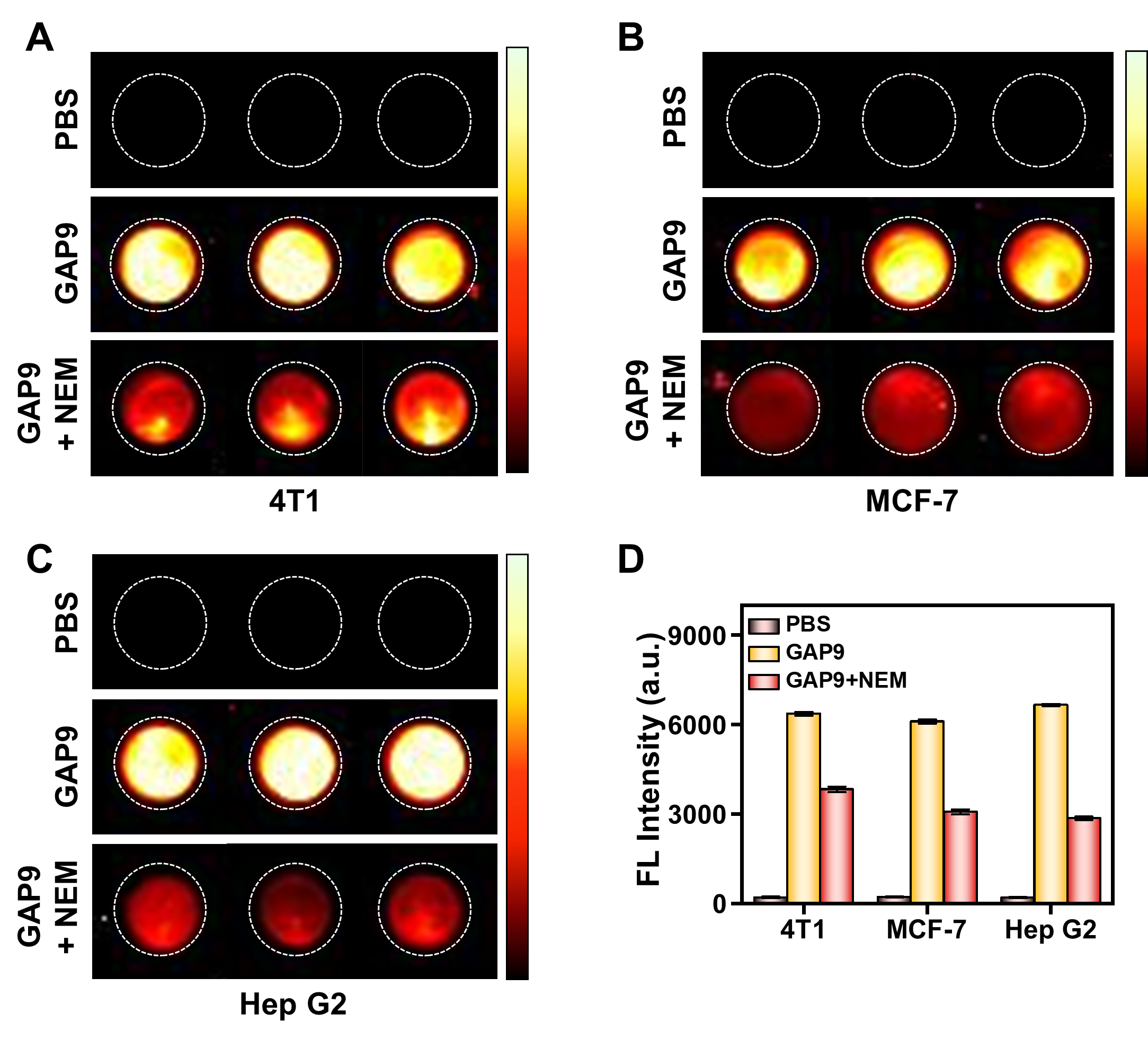


**Figure S20.** GSH-responsive performance of GAP9 on cancer cells. (A)-(C) NIR-II imaging of 4T1, MCF-7, and Hep G2 cells after different treatment with GAP9 (using LP1000 filter). (D) Quantitative luminescence intensity of 4T1, MCF-7 cells, and Hep G2 cells after different treatment with GAP9. All statistical data are presented as mean values ± SD. (n = 3 independent experiments).


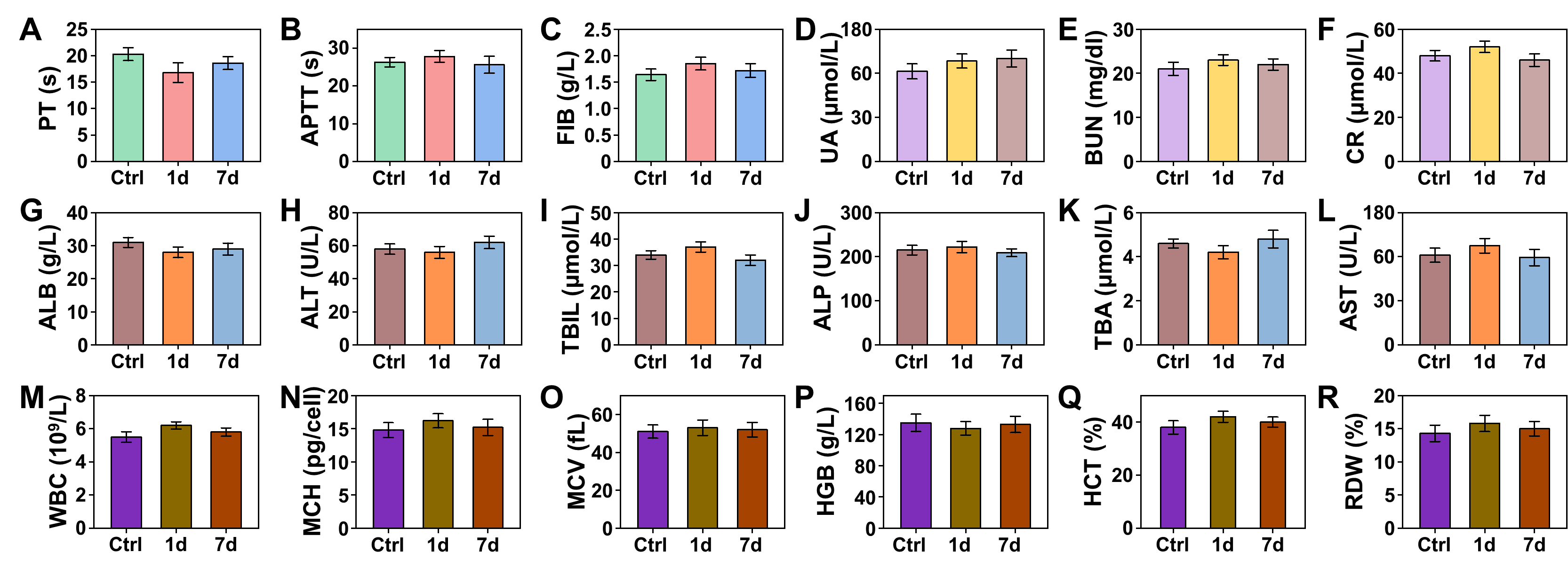


**Figure S21.** In vivo toxicity evaluation: blood biochemistry and hematology data of BALB/c mice treated with GAP9. The data were collected at different time point after intravenous injection: (A) prothrombin time (PT). (B) activated partial thromboplastin time (APTT). (C) fibrinogen (FIB). (D) urea (UA). (E) blood urea nitrogen (BUN). (F) creatinine (CR). (G) albumin (ALB). (H) alanine aminotransferase (ALT). (I) total bilirubin (TBIL). (J) alkaline phosphatase (ALP). (K) total bile acid (TBA). (L) aspartate aminotransferase (AST). (M) white blood cells (WBC). (N) mean corpuscular hemoglobin concentration (MCH). (O) mean corpuscular volume (MCV). (P) hemoglobin (HGB). (Q) hematocrit (HCT). (R) red cell distribution width (RDW). All statistical data are presented as mean values ± SD. (n = 3 independent experiments).


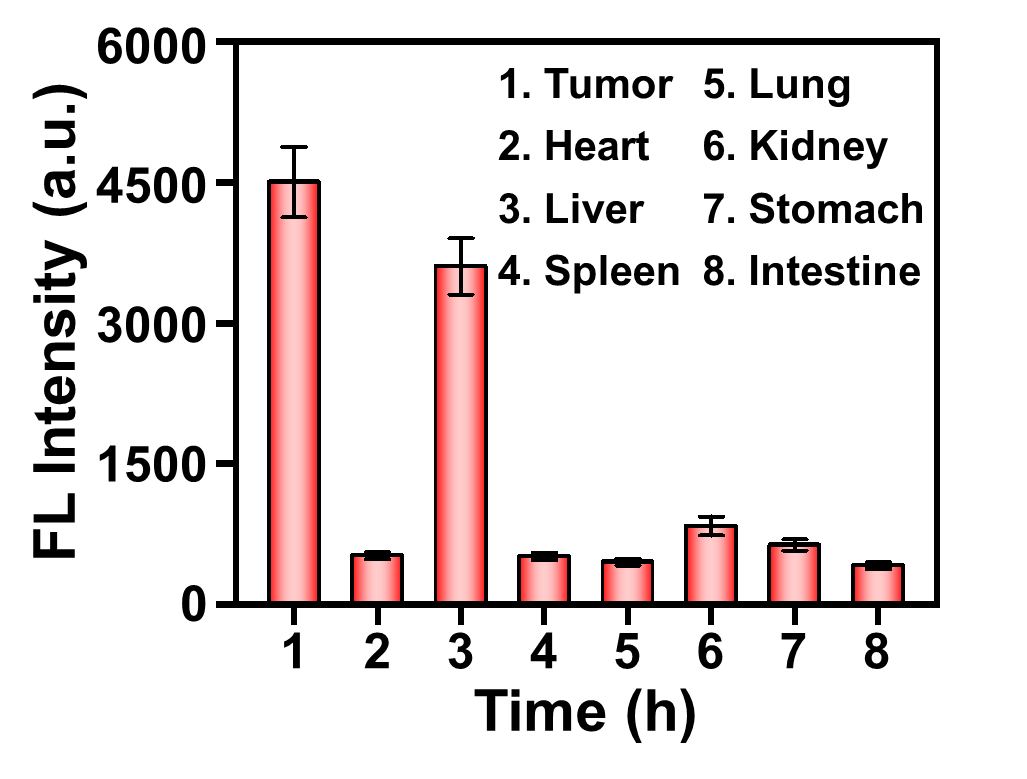


**Figure S22.** Corresponding statistical results of fluorescence intensity of major organs and tumors. All statistical data are presented as mean values ± SD. (n = 3).


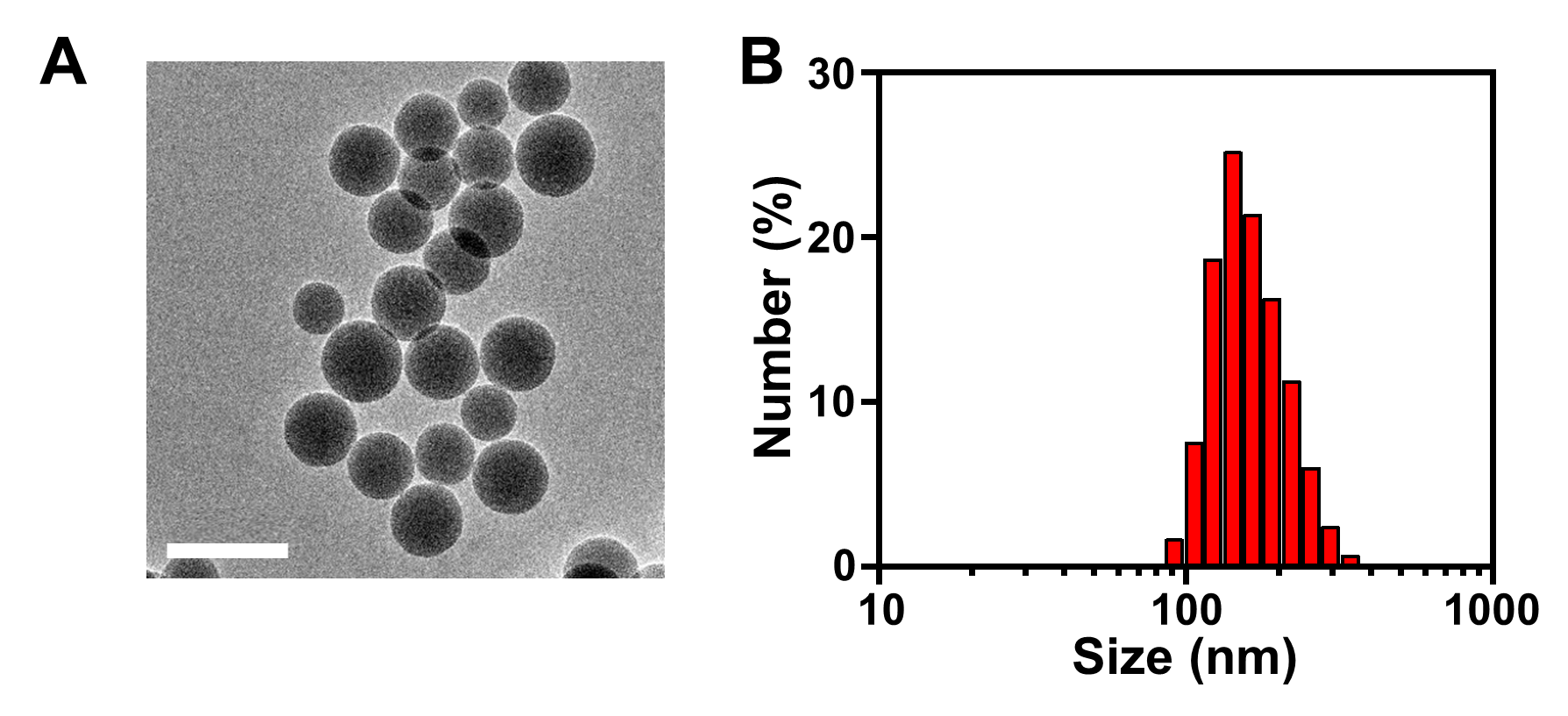


**Figure S23.** Characterization of IR820@SiO_2_. (A) Representative TEM images and (B) Hydrodynamic diameter of IR820@SiO_2_. Scale bars: 200 nm.


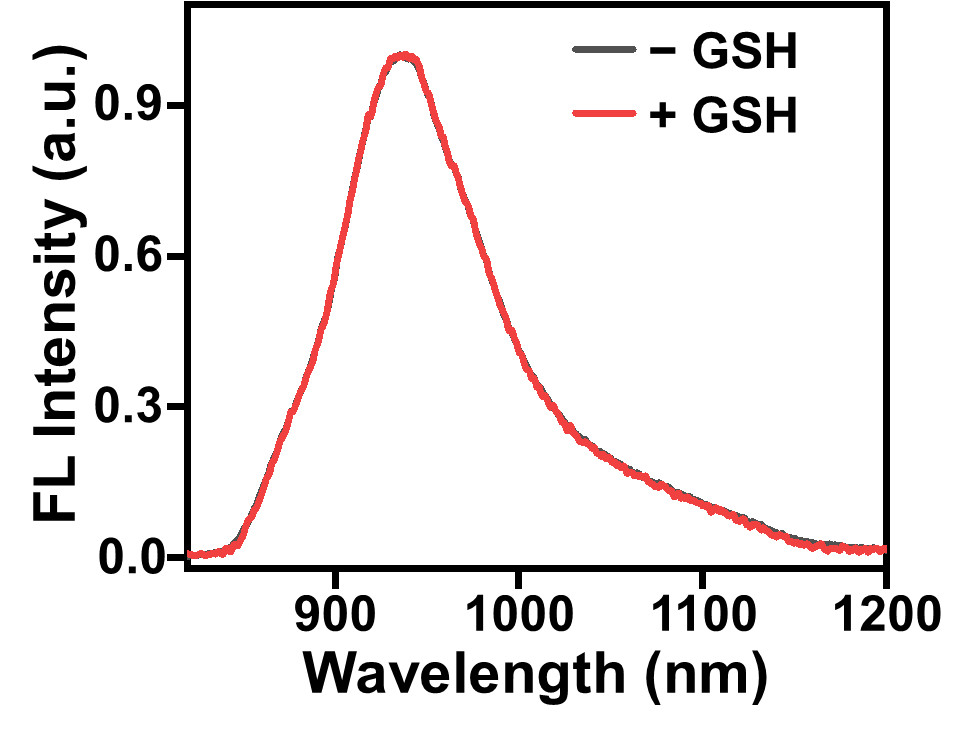


**Figure S24.** Fluorescence spectra of IR820@SiO_2_ in PBS (black lines) and PBS with GSH (10 mM, red lines), respectively.


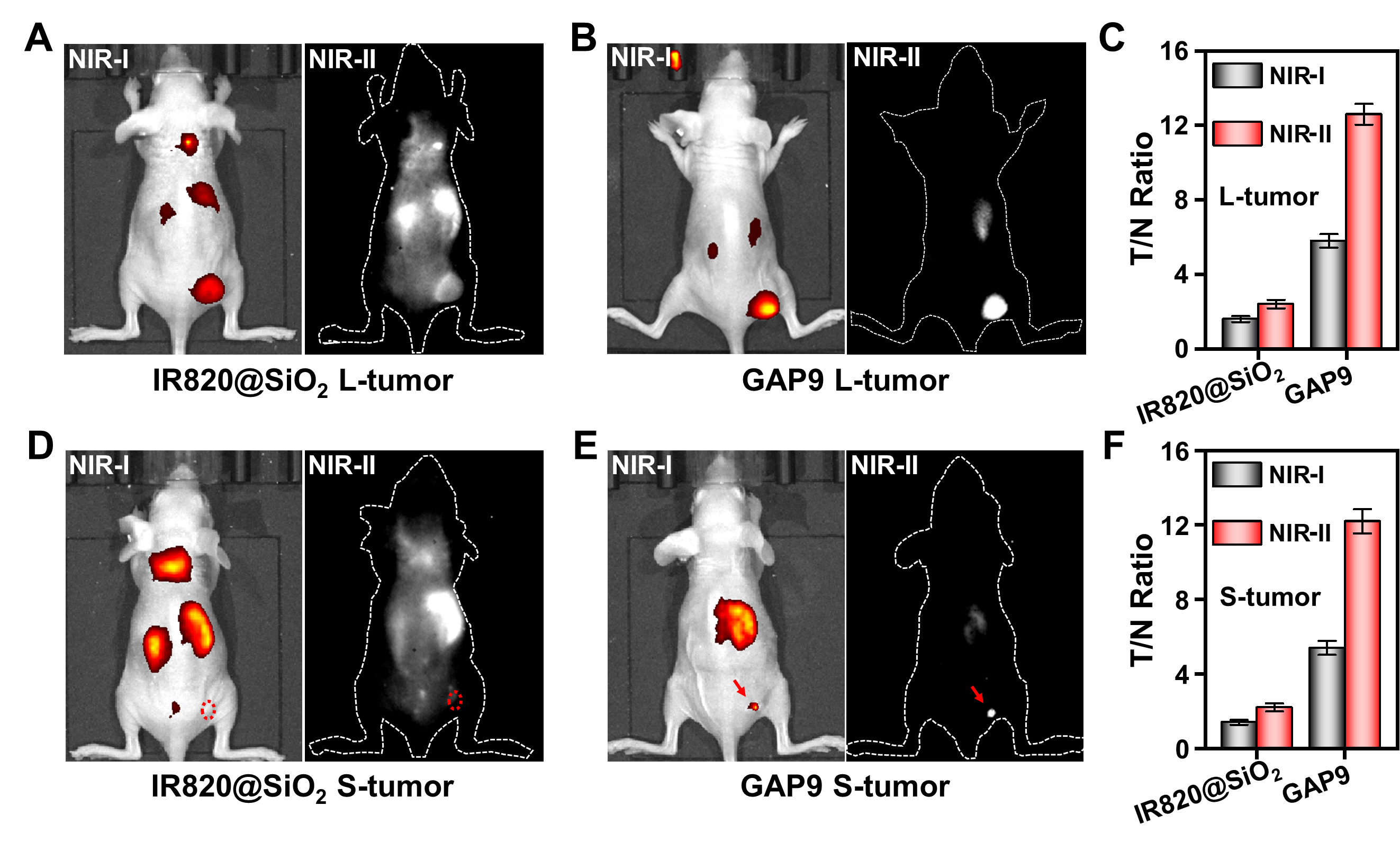


**Figure S25.** NIR-II imaging performance of GAP9 in living mice bearing subcutaneous 4T1 tumors with different sizes (Large: L, Small: S). Comparative NIR-I and NIR-II imaging in large-sized tumor models 6 h after intravenous injection of (A) IR820@SiO_2_ and (B) GAP9. (C) T/N ratio calculated from (A) and (B). Comparative NIR-I and NIR-II imaging in small-sized tumor models 6 h after intravenous injection of (D) IR820@SiO_2_ and (E) GAP9. (F) T/N ratio calculated from (D) and (E). Data represents the mean ± SD of triplicate measurements.


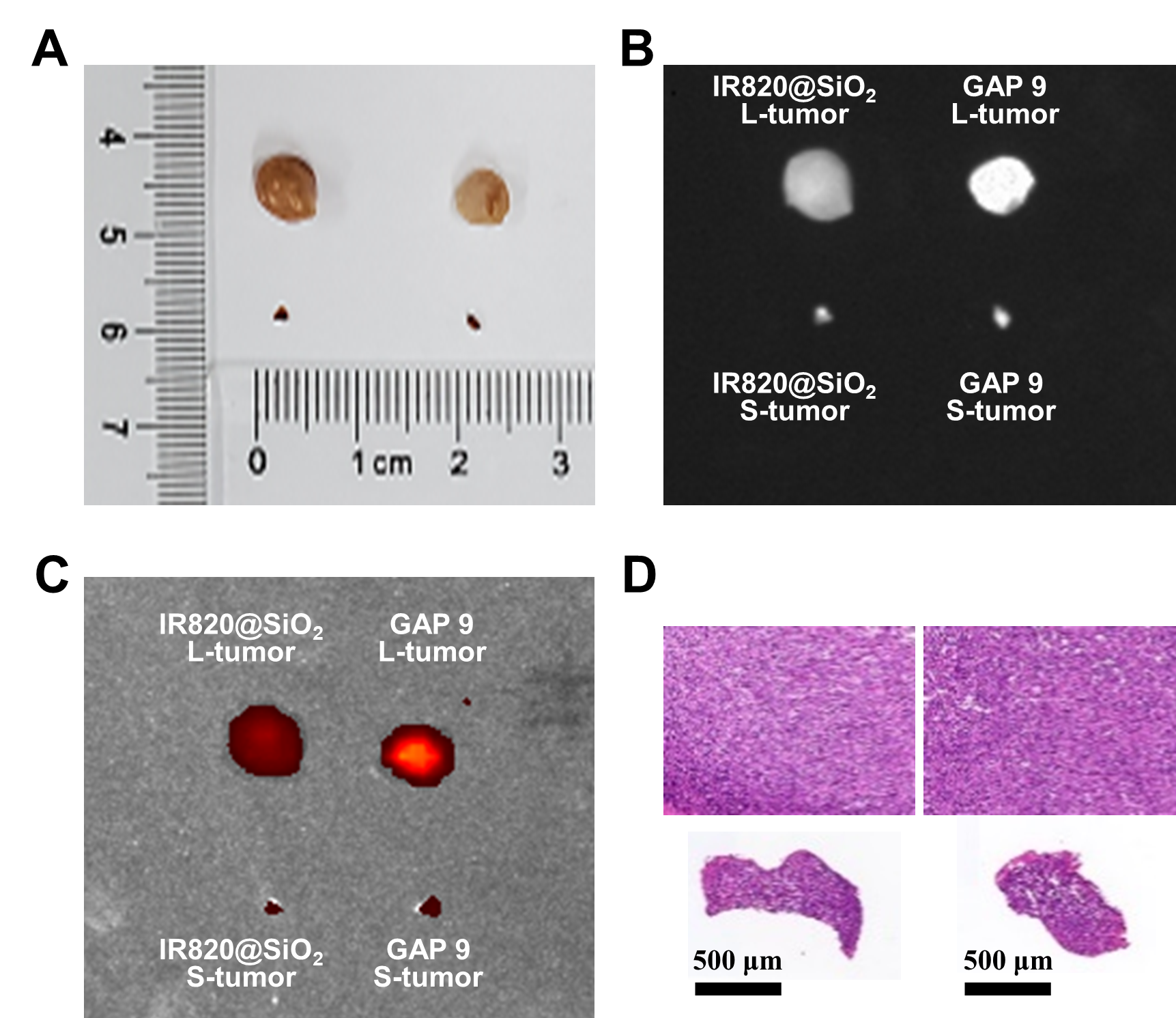


**Figure S26.** *Ex vivo* tumor imaging (A) White light imaging, (B) NIR-II fluorescence imaging, (C) IVIS imaging, (D) H&E staining of tumor tissues. Scale bars: 500 μm.

**3. References**

[1] Y. Geng, X. Guo, F. Yue, M. Xiang, Q. Zhu, "Mass Production of Multishell Hollow SiO_2_ Spheres With Adjustable Void Ratios and Pore Structures" *Adv. Mater.* **2024**, *36*, 2409421.
